# Multifocal Cerebral Microinfarcts Modulate Early Alzheimer’s Disease Pathology in a Sex-Dependent Manner

**DOI:** 10.3389/fimmu.2021.813536

**Published:** 2022-01-31

**Authors:** Sarah Lecordier, Vincent Pons, Serge Rivest, Ayman ElAli

**Affiliations:** ^1^ Neuroscience Axis, Research Center of CHU de Québec-Université Laval, Quebec City, QC, Canada; ^2^ Department of Psychiatry and Neuroscience, Faculty of Medicine, Université Laval, Quebec City, QC, Canada; ^3^ Department of Molecular Medicine, Faculty of Medicine, Université Laval, Quebec City, QC, Canada

**Keywords:** multifocal cerebral microinfarcts, Alzheimer’s disease, amyloid-beta (Aß), neuroinflammation, microglia, monocytes, cognition

## Abstract

Alzheimer’s disease (AD) constitutes a major cause of dementia, affecting more women than men. It is characterized by amyloid-β (Aβ) deposition and neurofibrillary tangles (NFTs) formation, associated with a progressive cognitive decline. Evidence indicates that AD onset increases the prevalence of cerebral microinfarcts caused by vascular pathologies, which occur in approximately in half of AD patients. In this project, we postulated that multifocal cerebral microinfarcts decisively influence early AD-like pathology progression in a sex dependent manner in young APP/PS1 mice. For this purpose, we used a novel approach to model multifocal microinfarcts in APP/PS1 mice *via* the sporadic occlusions of the microvasculature. Our findings indicate that microinfarcts reduced Aβ deposits without affecting soluble Aβ levels in the brain of male and female APP/PS1 mice, while causing rapid and prolonged cognitive deficits in males, and a mild and transient cognitive decline in females. In male APP/PS1 mice, microinfarcts triggered an acute hypoperfusion followed by a chronic hyperperfusion. Whereas in female APP/PS1 mice, microinfarcts caused an acute hypoperfusion, which was recovered in the chronic phase. Microinfarcts triggered a robust microglial activation and recruitment of peripheral monocytes to the lesion sites and Aβ plaques more potently in female APP/PS1 mice, possibly accounting for the reduced Aβ deposition. Finally, expression of Dickkopf-1 (DKK1), which plays a key role in mediating synaptic and neuronal dysfunction in AD, was strongly induced at the lesion sites of male APP/PS1 mice, while its expression was reduced in females. Our findings suggest that multifocal microinfarcts accelerate AD pathology more potently in young males compared to young females independently upon Aβ pathology *via* modulation of neurovascular coupling, inflammatory response, and DKK1 expression. Our results suggest that the effects of microinfarcts should be taken into consideration in AD diagnosis, prognosis, and therapies.

## Introduction

Alzheimer’s disease (AD) is a progressive neurodegenerative disorder that constitutes the most common form of dementia in the elderly (1, 2). AD patients display a wide range of clinical manifestations that comprise essentially progressive memory loss, executive function impairments, anxiety, depression, apathy, hallucination, agnosia, apraxia and aphasia (3–5). The neuropathology of AD is characterized by the deposition of amyloid-β (Aβ) plaques associated to Aβ accumulation, and the formation of neurofibrillary tangles (NFTs) associated to tau hyperphosphorylation, leading to brain atrophy caused by the loss of neurons and synapses (5–8). Deregulation of the innate immune response as well as the cerebrovascular functions are now well recognized as main components of AD etiology, pathogenesis and progression (9–11).

Inflammation associated with modulation of the activity of microglial cells and peripheral myeloid cells, namely monocytes, is critically involved in influencing Aβ pathology in AD (12). Accumulation of Aβ in the brain triggers microglial cell activation, which plays a double-edged sword in AD pathology (13), by promoting neuronal protection at the early stages *via* Aβ clearance (14, 15), and to neuronal loss at the advanced stages *via* release of neurotoxic and pro-inflammatory factors leading to chronic neuroinflammation (16, 17). Besides microglia, monocytes have been shown to be critically implicated in Aβ-mediated neuroinflammation (18, 19). Monocytes are mononuclear phagocytic cells that are regrouped in different subsets based on the expression level of surface markers, including lymphocyte antigen 6 complex, locus C (Ly6C) and C-X3-C motif chemokine receptor (CX3CR)1 (20). In rodents, the subsets comprise essentially the “classical” inflammatory Ly6C^high^ CX3CR1^low^ monocytes, which are actively recruited to the inflamed tissue to partake in the inflammatory response and differentiate into microglia-like cells, and the “non-classical” patrolling Ly6C^low^ CX3CR1^high^ monocytes, which actively patrol the vasculature to promote vascular system homeostasis and contribute as well to the early inflammatory response and tissue repair (19, 21). The exact role of Ly6C^low^ CX3CR1^high^ monocytes in various brain disorders, including AD, and their capacity to infiltrate the brain remain not fully elucidated. Nonetheless, CX3CR1^high^ monocytes have been shown to infiltrate the injured tissue to differentiate into regenerative macrophages that promote tissue neovascularization and repair *via* secretion of trophic and anti-inflammatory factors (22–24). Indeed, the Ly6C^low^ CX3CR1^high^ monocytes have been previously shown to play a critical role in mediating neuroprotection during excitotoxicity (25). Furthermore, Ly6C^low^ CX3CR1^high^ monocytes have been recently shown to contribute to the clearance of vascular Aβ micro-aggregates, attenuating Aβ pathology (18). Several reports have suggested that the Ly6C^low^ CX3CR1^high^ subset could arise from the Ly6C^high^ CX3CR1^low^ subset. In this regard, an intermediate Ly6C^int^ CX3CR1^high^ monocyte subset has been characterized and shown to integrate the characteristics of classical and non-classical monocyte subsets without possessing the vascular patrolling capabilities (22).

Various vascular factors have been shown to constitute significant risk factors for AD (26–30). Indeed, vascular dysfunction has been demonstrated to contribute to AD pathogenesis and progression by impairing cerebral blood flow (CBF), exacerbating Aβ and tau pathologies, triggering neuronal death, and inducing glial cell activation (31–35). The accumulating epidemiological, clinical, and experimental observations are highlighting the presence of an intimate pathological link between AD and cerebrovascular diseases (36). The vascular pathologies that comprise cerebral amyloid angiopathy (CAA) and cerebral small vessel disease (CSVD) have been shown to constitute the most prevalent forms of cerebrovascular diseases associated with AD (36, 37). Indeed, up to 42% of AD patients display cerebral microinfarcts caused by vascular pathologies (38–42). Despite the major efforts to investigate the role of various vascular risk factors on AD pathogenesis and progression, little is known about the impact of microinfarcts. The current state of knowledge suggests that vascular pathologies and AD-related neuropathology independently, but jointly, contribute to cognitive decline and dementia. For instance, some reports indicate that microinfarcts are very common, strongly correlating with dementia in very elderly AD patients (42, 43). Moreover, microinfarcts in patients with severe AD at advanced stages do not seem to influence the cognitive decline, which may be due to the overwhelming AD pathology (44). On the other hand, some other reports indicated that microinfarcts could exacerbate early AD pathology in younger patients (45, 46). These reports suggest that microinfarcts differentially influence AD pathology depending upon the disease stage. However, these are post-mortem clinico-pathological investigations, making it impossible to determine when microinfarcts occurred. Despite the high prevalence of microinfarcts in AD, the mechanisms underlying microinfarct-mediated modulation of AD pathology at the early stages remain elusive.

It is well evidenced in the literature that biological sex influences the prevalence and severity of AD as well as cerebrovascular diseases (47–51). Almost two third of patients living with AD are women who display poorer cognitive functions when compared to age-matching men (47–51). Furthermore, women have higher mortality rate and poor neurological recovery after cerebrovascular diseases (49–51). The mechanisms underlying these biological sex differences are still not fully understood, but the high longevity in females does not provide alone an explanation. This is supported by various reports indicating that female sex hormones provide protection for the brain of young women that is lost with age.

In this project, we aimed to investigate the impact of multifocal microinfarcts associated to CSVD in modulating early AD pathology in a sex-dependent manner. For this purpose, we used a novel mouse model of multifocal cerebral microinfarcts that was induced *via* the sporadic micro-occlusion of brain penetrating arteries in young male and female APP/PS1 mice. Our findings indicate that microinfarct induction in young APP/PS1 mice exacerbates cognitive decline and impairs neurovascular coupling in males, whereas in females those deficits were transient. Microinfarct induction unexpectedly attenuated Aβ pathology in males and females but triggered a robust microglial activation and recruitment of peripheral phagocytic immune cells, namely monocytes, more potently in the brain of females. Finally, we found out that Dickkopf-1 (DKK1), an endogenous inhibitor of the canonical Wnt pathway, which plays a major role in regulating synaptic transmission and plasticity (52), is strongly induced at the lesion sites of males compared to females upon microinfarct induction. Our study suggests that multifocal microinfarcts aggravate cognitive decline more potently in young APP/PS1 male mice compared to young females independently upon Aβ pathology *via* modulation of various mechanisms that include neurovascular coupling, neuroinflammation, and DKK1 expression. Our data indicate that the microinfarcts at the early stages of AD should be taken into consideration in the diagnosis and prognosis as well as in the development of tailored therapies.

## Materials and Methods

### Animal Experiments

APP/PS1 transgenic mice expressing the chimeric mouse/human Swedish amyloid precursor protein (APP695swe) and a mutant human presenilin1 (PS1-dE9) under the control of independent mouse prion promoter elements [B6.Cg-Tg(APPswe,PSEN1dE9)85Dbo/J] were used. C57BL/6J wildtype (WT) littermate mice were used as controls whenever necessary. CX3CR1^GFP/+^ mice in which an enhanced green fluorescent protein (EGFP) sequence replacing the first 390 bp of the coding exon (exon 2) of CX3CR1 gene allowing GFP expression in peripheral monocytes [B6.129P2(Cg)-Cx3cr1^tm1Litt/J^] were used to generate chimeric mice. Moreover, 5 months old male APP/PS1/CX3CR1^GFP/+^ triple transgenic mice, which were obtained by crossbreeding APP/PS1 x CX3CR1^GFP/+^ mice, were used to investigate the dynamics of CX3CR1^GFP/+^ cells in the brain of APP/PS1 mice. All animals were originally purchased from The Jackson Laboratory (Bar Harbor, ME, USA) backcrossed, maintained on a pure C57BL/6J background, and genotyped using the protocols recommended by The Jackson Laboratory. Five months old male and non-ovariectomized female mice were used. Young animals were chosen to better reflect the early phases of AD and the consequences of microinfarcts on disease progression. Mice were housed for a maximum of 5 per cage under standard laboratory conditions (23°C, 50-60% humidity, 12:12 hour light and dark cycle), and were provided with standard laboratory chow and water ad libitum. Mice were randomized for the experimental groups and neurobehavioral tests, and the experimenter was blind to the genotype as well as the experimental group. All animal procedures and handling were performed according to the Canadian Council on Animal Care guidelines, as implemented by the Laval University Animal Welfare Committee.

### Generation of Chimeric Mice

Generation of chimeric mice was performed as previously described (18, 53, 54). Briefly, femurs were flushed from CX3CR1^GFP/+^ donor mice in a sterile environment to obtain bone marrow cells. Cells were harvested using Dulbecco’s Phosphate Buffered Saline (DPBS; Wisent) and 5% fetal bovine serum (FBS). The extract was filtered using a 40 µm nylon filter and pelleted to remove any clumps. The cell pellet was collected with DPBS, then centrifuged and re-suspended in fresh DPBS. The recipient WT mice underwent a myelosuppressive chemotherapy regimen using busulfan (Otsuka America Pharmaceutical) and procytox (Baxter). The chemotherapy regimen was based on 2 injections per day (every 8 hours) of 10 mg/kg of busulfan (150 μl, intraperitoneally) for 4 days (a total of 80 mg/kg) followed by 1 injection per day of 100 mg/kg of procytox (150 μl, intraperitoneally) for 2 days (a total of 200 mg/kg). Mice were subcutaneously injected with 1 ml of 0.9% NaCl solution up to 1 week after procytox last injection to avoid dehydration. Mice were kept during the procedure in sterile cages and given previously irradiated food, and antibiotic treatment continued for 1 week following treatment. Chimerism was confirmed 8 weeks later by validating the presence of GFP^+^ myeloid cells in CD45^+^ cells in the blood circulation of CX3CR1^GFP/+^ → WT chimeric mice using flow cytometry to validate chimerism success, indicating that the bone marrow cells were reconstituted with monocytes expressing CX3CR1^GFP/+^ at 90% ± 1.78%.

### Induction of Multifocal Cerebral Microinfarcts

**Figure 1:**
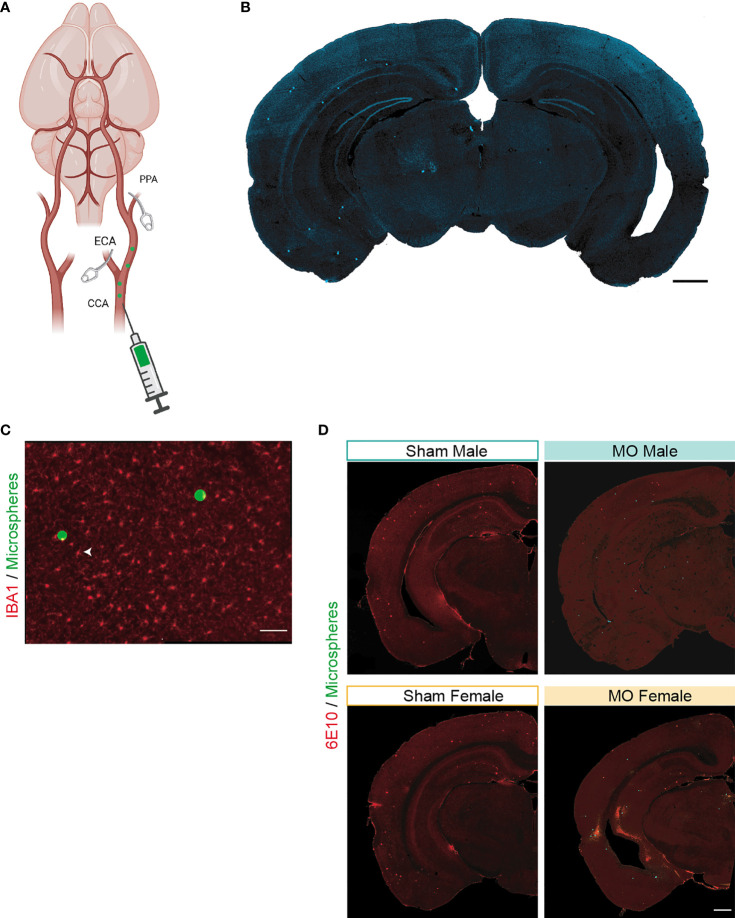
Surgical procedure to induce multifocal microinfarcts in the mouse brain. **(A)** A scheme illustrating the surgical procedure to induce sporadic microinfarcts in the mouse brain. The external carotid artery (ECA) and the pterygopalatine artery (PPA) were temporarily blocked using a microvascular clip. Sterilized FITC-tagged microspheres (n=2500; 20 µm) were slowly injected into the left common carotid artery (CCA). The ECA and PPA were unblocked after the injection of microspheres. The procedure was performed, as previously described (55). Created with BioRender.com. **(B)** A representative image of the fluorescent microspheres in a mouse brain. **(C)** A representative fluorescent image of sterile microspheres surrounded by non-reactive microglia labeled using IBA1 1 month after surgery. **(D)** Representative fluorescent images of 6E10 immunolabeled Aβ plaques (red) and microspheres (green) in the coronal brain sections of sham- and MO-operated male and female APP/PS1 mice. Scale bar = 500 µm **(B, D)** and 100 µm **(C)**.

Five months old male and female APP/PS1 mice, C57BL6/J mice, CX3CR1^GFP/+^ → WT mice and APP/PS1/CX3CR1^GFP/+^ mice were subjected to micro-occlusions (MO) to generate sporadic multifocal microinfarcts in the brain, as previously described (55). Briefly, mice were anesthetized under 1.5% isoflurane in 1.5 l/minute (95% O2) and body temperature was maintained between 36 and 37°C using a feedback-controlled heating system (Harvard Apparatus, QC, Canada). A midline neck incision was performed to expose the left common carotid artery (CCA), as well as the external carotid artery (ECA) and the pterygopalatine artery (PPA), which were temporarily blocked using a microvascular clip under a surgical microscope (Leica Microsystems, ON, Canada). Afterwards, 2500 sterilized FITC-tagged microspheres of 20 µm (Polysciences Inc., PA, USA) suspended in a 100 µl of PBS were slowly injected into the CCA using a 33G hypodermic needle (TSK Laboratory International, BC, Canada). The microspheres of 20 µm have been shown to allow occlusion of penetrating cerebral arterioles and capillaries (**Figure 1A**) (55). The ECA and PPA were temporarily blocked prior to injection to ensure that all microspheres are directed towards the brain. After injection, the ECA and PPA were unblocked, the needle was gently removed from the CCA and the bleeding was immediately stopped by applying pressure using bioabsorbable Gelfoam (Pfizer, NY, USA). APP/PS1 mice were euthanized 1 month following induction of surgery (mice were 6 months old), while CX3CR1^GFP/+^ → WT mice were divided into 3 subgroups that were sacrificed 3 days, 1 week and 1 month post-surgery. APP/PS1/CX3CR1^GFP/+^ mice were sacrificed 1 week post-surgery. Sham-operated mice underwent the same surgical procedure without microsphere injection. Distribution analysis of the microspheres showed that 9% were present in the hippocampus, 4% in the white matter, 5% in the striatum, 31% in the thalamus, 34% in the neocortex and 15% in other structures, producing microinfarcts in 7% of cases in the hippocampus, 5% in the white matter and striatum, 3% in the thalamus, 1% in the cortex, as previously described (**Figure 1B**) (55). The fluorescent microspheres are sterile and non-immunogenic, outlined by the absence of any direct impact on microglial reactivity *per se* (**Figure 1C**). Finally, the injected microspheres were distributed throughout the brain of APP/PS1 mice in which Aβ pathology is developing (**Figure 1D**).

### Neurobehavioral Analysis

Animals underwent neurobehavioral tests prior to surgery (baseline) and at different time points post-surgery, as follow:

*Novel object recognition (NOR) test*: The test was used to assess recognition memory, as previously described (18, 52, 56). Briefly, in an open arena (45 cm x 45 cm) the mouse was free to explore two identical objects for 10 minutes in an acquisition phase. The animal was placed back to its home cage for 1 hour, before the 10 minutes test phase. In this recall phase, one of the objects was replaced by a new one. We evaluated the time spent on both objects to assess mice performances. Indeed, mice are very curious and attracted to novelty which will lead to a higher exploratory time on the novel object during the test phase. Trials were recorded by a camera placed above the arena and a blind experimenter scored by hand the collected videos. The arena and objects were cleaned with 70% alcohol between each mouse and trial. The task was performed at baseline, 1 week and 1-month post-surgery. A discrimination index ratio was calculated as follow: time novel object/(time novel object - time familiar object). A ratio = 0.5 means an equal exploration of the 2 objects, a ratio > 0.5 means a higher exploratory time for the novel object, and < 0.5 means a preference for the familiar object.

*Open field (OF) test:* The test was used to assess anxiety and general ambulatory ability as previously described (57). Briefly, in an open arena (45 cm x 45 cm) placed under a camera controlled by a computer, the mouse was free to move for 10 min. Assessment of the animals’ movements were recorded, tracked, and analyzed with the Anymaze software on which the arena was divided into different zones namely, the center, borders, and corners. Parameters evaluated were, the time spent in the center as it is at risk for mice that are prey and the distance moved. An increase in the time spent in the center would indicate a disinhibitory behavior whereas a decrease shows an anxious state. A decrease in the distance could either mean a freezing state, a typical anxiety behavior, or motor impairment.

### Laser Speckle Contrast Imaging Analysis

Laser Speckle contrast imaging (LCSI) was used to evaluate the longitudinal variation of CBF following the induction of microinfarcts. LSCI was performed 24 hours after induction of microinfarcts to evaluate the impact on CBF in the acute phase, and 1 month afterwards in the same animals to assess the long-term consequences. Mice were anesthetized with ketamine/xylazine (1 mg/10 ml) and received 1 ml of Ringer’s lactate solution. Prior to surgery the head was shaved, lidocaine/bupivacaine solution applied on the incision sites (100 µl) and the ears (50 µl/ear), and the skin disinfected. MO- and sham-operated mice were placed on a stereotaxic frame (RWD Life Science Inc., CA, USA), the skull was then exposed by removing the skin using fine-tip forceps, and relative CBF was measured using 2D high-resolution laser blood flow imager (OMEGAZONE OZ-3, OMEGAWAVE, INC.). The OZ-3 system is equipped with a visible and near infra-red (NIR) CCD camera, which allows showing real images continuously, and to compare the color difference between real and blood flow images. Furthermore, the system is equipped with a measurement and analysis software that allows simultaneous quantification of the relative CBF in different regions of interest (ROI). The mean of relative CBF of each hemisphere in similar ROI was quantified, and a ratio of ipsilateral/contralateral values were computed. A ratio of 1 indicates that the relative CBF values are equal in both hemispheres, and a ratio below 1 would indicate a reduced CBF in the ipsilateral, and a ratio above 1 would outline a CBF overall deregulation.

### Tissue Sample Preparation

Male and female WT and APP/PS1 mice were sacrificed 1-month post-surgery, while male and female CX3CR1^GFP/+^ → WT mice were divided into 3 subgroups that were sacrificed 3 days, 1 week and 1 month post-surgery. Male APP/PS1/CX3CR1^GFP/+^ mice were sacrificed 1 week post-surgery. Mice were deeply anesthetized with a solution containing ketamine (90 mg/ml) and xylazine (10 mg/ml) and sacrificed *via* intracardiac perfusion with 0.9% NaCl. For immunohistochemical analysis, the brains were retrieved, post-fixed in 4% paraformaldehyde (PFA) for 24 hours and transferred into 20% sucrose containing 4% PFA for a minimum of 24 hours. Brains were then cut into 25 μm coronal sections using a freezing microtome (Leica Biosystems, ON, Canada), and serial sections were collected in 12 well-plates in an antifreeze solution (30% glycerol, 30% ethylene glycol in 0,9% NaCl, phosphate buffer (PB)) and kept at -20°C for further use. For Western blot analysis, the brains were removed and dissected to separate the hippocampus and cortex from each hemisphere that were snap-frozen on dry ice and kept at -80°C until further use. Each region was then homogenized separately in a NP40 lysis buffer containing 1% of phosphatase inhibitors and 1% of protease inhibitors, sonicated, and then stored in -80°C until further use.

### Immunofluorescence Analysis

Free floating brain sections were rinsed 3x with potassium phosphate buffer saline (KPBS) and incubated at room temperature in a blocking/permeabilization solution containing 4% normal goat serum (NGS), 1% BSA, 1% Triton X-100 in KBPS for 45 minutes. Brain sections were then incubated with primary antibodies diluted in KPBS solution containing 1% NGS, 1% BSA, 0.2% Triton X-100 overnight at 4°C. The following primary antibodies were used; anti-Aβ (6E10) (1:1000; mouse anti-human, Biolegend, 8030001), anti-ionized calcium binding adaptor molecule (IBA)-1 (1:1000, rabbit anti-mouse, WAKO, 019-19741), anti-cluster of differentiation (CD)-68 (1:1000, rat anti-mouse, Bio-Rad, Hercules, CA, USA, MCA1957) and anti-CD45 (1:500, rat anti-mouse, BD bioscience, 55376) and anti-nuclear receptor subfamily 4 group A member 1 (Nr4A1 or Nurr77) (1:250, rabbit, Abcam, ab13851). The next day, brain sections were rinsed 3x with KPBS, and incubated for 2 hours at room temperature in the dark with one of the following Cy3 or Cy5-conjugated secondary antibody; AffiniPure goat anti-rabbit IgG (H+L) (1:1000, Invitrogen, A10523) and AffiniPure goat anti-mouse IgG (H+L) (1:1000, Jackson Immunoresearch, 115-165-003); AffiniPure goat anti-rat IgG (H+L) (1:1000, Jackson Immunoresearch, 112-175-143). The brain sections were then rinsed 2x with KPBS and incubated with 4′,6-diamidino-2-phenylindole (DAPI) (1:10 000) for 5 minutes. The brain sections were mounted onto Superfrost^®^ Plus slides and cover-slipped with Fluoromount-G^®^ anti-fade medium (Sigma-Aldrich). Epifluorescence images were taken using Axio Observer microscope equipped with a module for optical sectioning (Apotome.2) and Axiocam 503 monochrome camera, and processed using ZEN Imaging Software (Carl Zeiss Canada, Toronto, ON, Canada). The density of IBA1 and CD68 immunolabeled cells as well as the number and volume of 6E10 immunolabeled Aβ plaques were assessed using unbiased computer-assisted stereological software (Stereologer; SRC Biosciences, FL, USA) (58).

### Immunohistochemical Analysis

Free floating brain sections were processed as specified before, and then incubated with a biotinylated goat anti-mouse Immunoglobulin G (IgG; H+L) (1:1000, Vectorlab, BA-9200). The next day, the brain sections were rinsed 3x with KPBS, and then incubated for 30 minutes at room temperature with Avidin-Biotin Peroxidase Complex (ABC) (Vectastain Elite Kit Standard). Brain sections were rinsed 3x with KPBS before incubation with 3,3’ 3,3′-diaminobenzidine tetrahydrochloride (DAB; Sigma-Aldrich), washed 3x with KPBS, and dried overnight at room temperature. The next day, brain sections were mounted onto Superfrost^®^ Plus slides and dehydrated *via* immersion in an increased concentration of ethanol (EthO) solution (50%, 70%, 75%, 95% and 100%), and finally immersed in Xylene solution 2x times for 3 minutes. The slides were then scanned, and the intensity of DAB signal in the ipsilateral as well as contralateral (i.e. background) cortex and hippocampus was analyzed using ImageJ software, as previously described (52). Ratio of intensity ipsilateral/contralateral was computed and values above 1 indicates an increased IgG intensity (i.e. infiltration) in the ipsilateral brain structures.

### Thioflavin S Staining

Free floating brain sections were rinsed 3x with KPBS, mounted onto Superfrost^®^ Plus slides, and dried overnight at room temperature prior to staining. The next day, the mounted slides were immersed in a cold 4% PFA solution for 45 minutes to fix the brain sections. The slides were then rinsed in KPBS for 10 minutes and incubated next for 1 hour in a 1% Thioflavin S solution diluted in MilliQ water (Millipore Sigma, ON, Canada). The slides were washed 2x in 100% EthO for 1 minutes and next in 80% EthO for 1 minute, and finally 3x in MilliQ water for 1 minutes. The slides were dried overnight and coverslipped with Fluoromount-G^®^ anti-fade medium (Sigma-Aldrich). The overall number of Thioflavin S-labeled vessels in the brain of APP/PS1 mice was counted using the fluorescence Axio Observer microscope (Carl Zeiss Canada).

### RNAscope™ Fluorescent *In Situ* Hybridization

Free floating brain samples were mounted onto Superfrost^®^ Plus slides and kept at -20°C to dry for 1 hour. The mounted slides were incubated for 15 minutes in 4% PFA at 4°C to fix the brain sections and washed with 0.1M PBS. The brain sections were dehydrated using EthO, and a hydrophobic barrier was drawn around the sections. RNAscope^®^ Fluorescent Multiplex Reagent Kit was used to perform the experiments following the manufacturer’s recommendations (Advanced Cell Diagnostics, Newark, CA, USA). Five drops of Protease III (provided in the kit) were added to entirely cover the brain sections, which were incubated at room temperature for 40 minutes. Following a series of washes with 0.1M PBS, 4 drops of mouse *DKK1* probe (Advanced Cell Diagnostics) were added to entirely cover each brain section, and the slides were placed into a slide rack, and inserted in the humidity control tray of the HybEZ™ Oven for 2 hours at 40°C. The humidity control tray was removed from the HybEZ™ Oven, one slide at a time, and the excess of liquid was removed and rinsed with a 1X washing buffer. Four drops of Amp-1-FL were added to entirely cover each brain section, which were incubated again in the HybEZ™ Oven for 30 minutes at 40°C. This process was repeated with Amp-2-FL, Amp-3-FL, and Amp-4-FL-Alt B. Finally, 4 drops of DAPI (provided in the kit) were added to each brain section and kept for 30 seconds, followed by a series of washes with 1X washing buffer. The slides were next mounted with 80 μl of Fluoromount-G^®^ anti-fade medium, carefully cover-slipped, and placed at 4°C in the dark until analysis under the fluorescence Axio Observer microscope (Carl Zeiss Canada).

### Western Blot Analysis

Twenty µg of proteins harvested either from the cortex or the hippocampus were mixed with 1X sodium dodecyl sulfate (SDS)-loading buffer and heated for 10 minutes at 95° C. Samples were run on 8–16% gradient precast acrylamide gels (Bio-Rad) and subjected to electrophoresis using Criterion^®^ cell (Bio-Rad). Following migration, resolved protein bands were transferred onto a 0.45 µm polyvinylidene fluoride (PVDF) membrane (EMD Millipore) for 30 minutes at 200V on ice using Criterion^®^ blotter (Bio-Rad). The PVDF membranes were washed 3x with a 0.1M tris buffer saline (TBS) solution containing 0.5% Tween-20 (TBS-T; Sigma-Aldrich) for 10 minutes and blocked in TBS-T with 5% (w/v) skim milk for 30 minutes at room temperature. The PVDF membrane was then incubated overnight at 4°C with different primary antibodies diluted at 1:1000 in TBS-T solution. The following primary antibodies were used; mouse anti-rabbit ATP Binding Cassette Subfamily B Member 1 (ABCB1) (1:1000, Santa Cruz Biotechnology, sc-8313), mouse anti-rabbit LDL Receptor Related Protein 1 (LRP1) (1:1000, Abcam, AB-92544), mouse anti-rabbit beta-site APP cleaving enzyme 1 (BACE1) (1:1000, Abcam, ab108394), and anti-mouse actin (1:1000, EMD Millipore, MAB 1501). Primary antibodies were detected with the appropriate horseradish peroxidase (HRP)-conjugated secondary antibodies (Jackson Immunoresearch, West Grove, PA, USA) that were diluted 1:5000 in TBS-T and revealed by enhanced chemiluminescence plus (ECL) solution (Bio-Rad). β-actin were used to ensure equal protein loading. Blots were revealed using Clarity western (ECL) substrate (Bio-Rad) and digitized using Biorad chemidoc XRS+ (Biorad, Montreal, Qc, Canada). Digitized blots were densitometrically analyzed with ImageJ software corrected for protein loading by means of β-actin, and expressed as relative values comparing different groups, as described (58).

### Soluble Human Aβ ELISA Analysis

The levels of soluble Aβ_40_ and Aβ_42_ in the contralateral and ipsilateral hemisphere of mice were quantified using Human Amyloid β 1-42 and 1-40 ELISA kits (EMD Millipore, EZHS SET). The experimental procedure for the detection of Aβ_42_ and Aβ_40_ was performed as recommended by the manufacturer’s instructions. Absorbance was obtained using a microtiter plate reader (SpectraMax 340PC, Molecular Devices), and analyzed using SOFTmax Pro3.1.1 software (Molecular Devices), as previously described (18).

### Flow Cytometry Experiments

Blood samples were collected from the submandibular vein into ethylene-diamine-tetra-acetic acid (EDTA) coated vials (Sarstedt, Montréal, QC, Canada) for the analysis of circulating monocytes and neutrophils. The experimental procedure was performed as previously described (59). Briefly, 50 μl of total blood was incubated at room temperature for 20 minutes with 1 ml of ACK (ammonium, chloride, potassium) lysing buffer to get rid of red blood cells. Samples were then washed with 3 ml of DPBS and centrifuge at 400 g for 8 minutes à 4°C. The supernatant was removed, and cells were resuspended with 100 µl of blockage solution, 1 µl CD16/CD32 antibody (BD bioscience) diluted in 100 µl of DPBS per tube and incubated for 10 min on ice. Then, 100 µl of antibody mix added in each tube which comprises cluster of differentiation (CD)-45 FITC (1:100, BD Pharmigen, 553079), cluster of differentiation (CD)-11b Pe-Cy7 (1:100, EBioscience, 25-0112), Ly6C V450 (1:100, BD Bioscience, 560594), Ly6G Pe (1:100, BD bioscience, 551461) and live/dead (1:50, Life technologies, L23105) for 30 minutes on ice in the dark. The remaining cells were next washed with 3 ml DPBS, centrifuged for 8 minutes at 400 g, resuspended in 300 µl of DPBS, and complemented with 50 µl of 123count eBeads™ Counting Beads (Invitrogen, 01-1234-42). Samples were processed using a LSR II flow cytometer, and data was acquired using BD FACS Diva software (Version 6.1.2, BD Bioscience), and analyzed using FlowJo software v10 (Tree Star; Ashland, OR, USA).

### Statistical Analysis

Data are presented as the mean ± standard error of the mean (SEM). For comparison between the 2 main different experimental groups, data was analyzed using standard two-tailed unpaired *t*-test. For ratio analysis, data was analyzed using a one sample *t*-test to compare the mean of experimental groups with 0.5 that represents a probability ratio of 50%. P-value *<* 0.05 was considered statistically significant (95% confidence interval). Statistical analyses were carried out using the GraphPad Prism Version 8.0 for Mac OS X (GraphPad Software).

## Results

### Microinfarcts Attenuates Early Aβ Pathology in APP/PS1 Mice in a Sex-Dependent Manner

**Figure 2:**
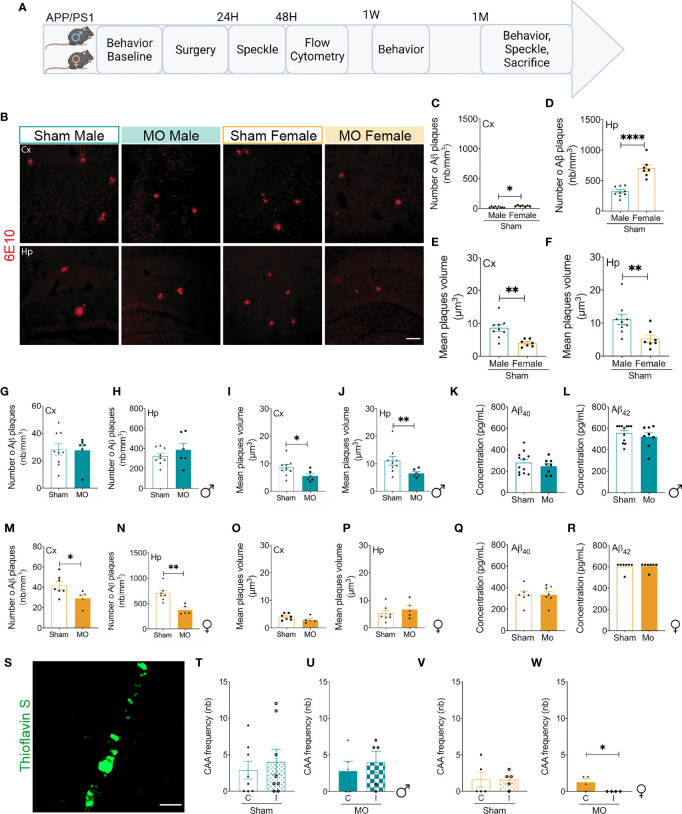
Modulation of Aβ pathology in young APP/PS1 mice in response to microinfarcts. **(A)** A scheme illustrating the experimental design of our study. Created with BioRender.com. **(B)** Representative fluorescent images of 6E10 immunolabeled Aβ plaques (red) in the cortex and hippocampus of sham- and MO-operated male and female APP/PS1 mice. Immunofluorescence combined to stereological analysis shows that sham-operated female APP/PS1 mice exhibit a higher number of Aβ plaques in the **(C)** cortex and **(D)** hippocampus compared to sham-operated males, while sham-operated male APP/PS1 mice develop Aβ plaque of higher volume compared to sham-operated females in the **(E)** cortex and hippocampus **(F)**. Moreover, immunofluorescence combined to stereological analysis indicates that the number of Aβ plaques in the **(G)** cortex and **(H)** hippocampus of MO-operated male APP/PS1 mice remains unchanged, but the volume of Aβ plaques decreased in MO-operated mice in the cortex **(I)** and hippocampus **(J)**. ELISA analysis shows that the levels of soluble **(K)** Aβ_40_ and **(L)** Aβ_42_ in the brain of male APP/PS1 mice remain unchanged. Immunofluorescence combined to stereological analysis shows that the number of Aβ plaques decreases in the **(M)** cortex and **(N)** hippocampus of MO-operated female APP/PS1 mice compared to sham-operated animals, whereas the volume of Aβ plaques remains unchanged in both structures **(O, P)**. ELISA analysis indicates that the levels of soluble **(Q)** Aβ_40_ and **(R)** Aβ_42_ remain as well unchanged in the brain of MO-operated female APP/PS1 mice. Histological analysis of Thioflavin labeling **(S)** shows that the frequency of CAA (Thioflavin S^+^; green) remains unchanged in the ipsilateral hemisphere of **(T)** sham-operated APP/P1 mice and **(U)** MO-operated APP/PS1 mice compared to the contralateral hemisphere, whereas it remains unchanged in the brain of **(V)** sham-operated female APP/PS1 mice, and **(W)** decreases in the ipsilateral hemisphere of MO-operated female APP/PS1 mice. Data are mean ± SEM (n = 5-10 animals/group). *P < 0.05/**P < 0.01/****P < 0.0001 compared to sham-operated WT or APP/PS1 mice (two-tailed unpaired t-test). Scale bar = 250 µm **(B)** and 500 µm **(S)**.

Deposition of Aβ in the brain constitutes one of the key pathological hallmarks of AD (8). In APP/PS1 mice, AD-like pathology is associated with Aβ depositions and cognitive decline that begin around the age of 4 months, progressing over time to become apparent in histological and neurobehavioral tests around 6 months of age (60–62). Although microinfarcts have been shown to occur at the early stages of AD, the consequences on Aβ pathology remain unclear. As such, we investigated the impact of multifocal cerebral microinfarcts induced using MO on early Aβ pathology in young male and female APP/PS1 mice to better appreciate the progression of AD-like pathology before it gets overwhelmed with advanced age (**Figure 2A**). For this purpose, brain sections were immunolabeled with a 6E10 antibody that recognize human Aβ and were processed for stereological analysis. Interestingly, we found out that the overall baseline density of Aβ plaques was lower in the cortex and hippocampus of sham-operated male APP/PS1 mice in comparison to females (**Figures 2B–D**), whereas the mean volume of individual Aβ plaques was more important in the cortex and hippocampus of sham-operated male APP/PS1 mice in comparison to females (**Figures 2B, E, F**). This observation suggests that Aβ pathology is fundamentally different in male and female APP/PS1 mice, with males having a lower number of large Aβ plaques, while females exhibiting a higher number of relatively small Aβ plaques. Furthermore, our analysis indicated that the density of Aβ plaques remained unchanged (**Figures 2B, G, H**), whereas the mean volume of individual Aβ plaques was decreased (**Figures 2B, I, J**), in the cortex as well as the hippocampus of MO-operated male APP/PS1 mice. The early induction of multifocal microinfarcts did not influence the levels of soluble Aβ_40_ and Aβ_42_ in the brain of male APP/PS1 mice (**Figures 2K, L**). On the other hand, the density of Aβ plaques was reduced (**Figures 2B, M, N**), whereas the mean volume of individual Aβ plaques did not change (**Figures 2B, O, P**), in the cortex as well as the hippocampus of MO-operated female APP/PS1 mice. As observed in males, multifocal microinfarct induction did not affect the levels of soluble Aβ_40_ and Aβ_42_ in the brain of MO-operated female APP/PS1 mice (**Figures 2Q, R**). The analysis suggests that microinfarcts attenuate parenchymal Aβ pathology by reducing the size of individual Aβ plaques in the brain of male APP/PS1 mice, while decreasing the overall number of Aβ deposits in the brain of females. We next evaluated the impact of multifocal microinfarcts on the frequency of CAA associated with vascular Aβ deposition in the brain of male and female APP/PS1 mice by examining the number of Thioflavin S-labeled arteries (**Figure 2S**). No changes were detected in the frequency of CAA in the brain of sham-operated (**Figure 2T**) and MO-operated (**Figure 2U**) male APP/PS1 mice. Similarly, the frequency of CAA in the brain of sham-operated female APP/PS1 mice remained unchanged (**Figure 2V**), whereas it was decreased in MO-operated mice (**Figure 2W**). Our results suggest that multifocal microinfarcts attenuated early Aβ pathology in APP/PS1 mice in a sex-dependent manner by differentially reducing the density and volume of parenchymal Aβ plaques as well as deposition of vascular Aβ aggregates.

### Cognitive Functions Are Sex-Dependently Impaired After Multifocal Microinfarction

**Figure 3:**
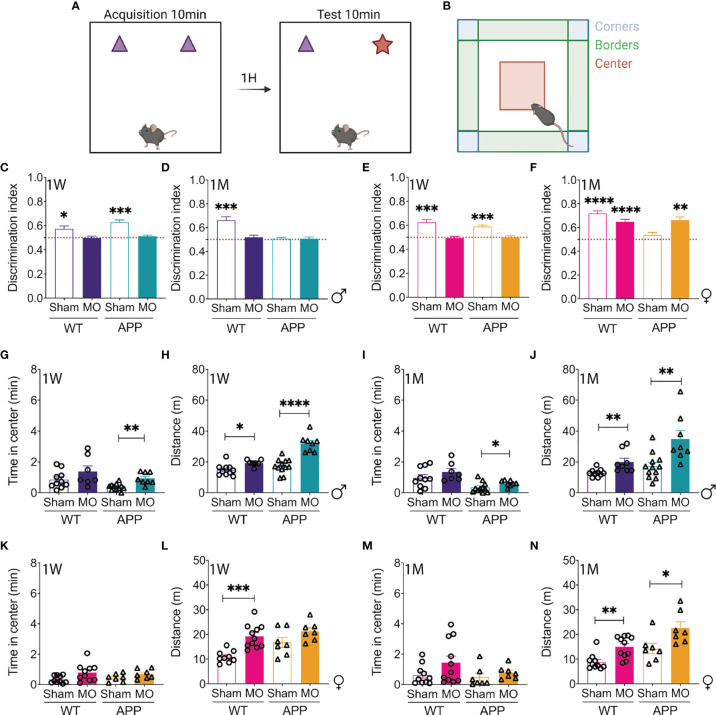
Multifocal microinfarction impairs the cognitive functions of APP/PS1 mice in a sex-dependent manner. A scheme illustrating the **(A)** Novel Object recognition (NOR) test and **(B)** open field (OF) test. Created with BioRender.com. Analysis of the discrimination index ratio indicates that **(C)** sham-operated male WT as well as APP/PS1 mice spend more time exploring the novel object during the test phase whereas MO-operated WT and APP/PS1 spend equal time in exploring both objects, 1 week post-microinfarct induction. At 1 month post-microinfarct induction, **(D)** sham-operated male WT mice recognize the novel object during the test phase, while sham-operated male APP/PS1 mice as well as MO-operated male WT and MO-operated APP/PS1 mice spend equal time exploring both objects. Analysis of the discrimination index ratio reveals that **(E)** sham-operated WT and APP/PS1 animals explore more the novel object, in contrast to MO-operated WT and MO-operated APP/PS1 mice that spend equal time exploring both objects, 1 week post-microinfarct induction. At 1 month post-microinfarct induction, **(F)** sham-operated female WT mice, MO-operated female WT mice and MO-operated female APP/PS1 spend more time exploring the novel object, while the sham-operated female APP/PS1 mice spend equal time exploring both objects. Analysis of the time spent in the center of the OF and the distance moved in the maze indicate that **(G)** MO-operated male APP/PS1mice spend more time in the center associated with an **(H)** increased distance travelled in the maze, while MO-operated male WT mice show only an increase in the distance travelled, 1 week post-microinfarct induction. **(I)** MO-operated male APP/PS1 mice still spend more time in the center of the maze compared to the sham-operated male APP/PS1 mice associated with **(J)** an increase in the distance travelled, while the MO-operated male WT littermate mice only exhibit an increase in the distance travelled. Analysis of the same parameters in females show that **(K)** no changes are noted in the time spent in the center of the maze while **(L)** an increase in the distance travelled is observed in MO-operated WT mice, 1 week post-microinfarct induction. At 1 month post-microinfarct induction, **(M)** no changes are noted in the time spent in the center of the OF. However, **(N)** an increase in the distance travelled is observed in MO-operated WT mice and APP/PS1 mice compared to sham-operated mice. Data are mean ± SEM (n = 5-10 animals/group). *P < 0.05/**P < 0.01/***P < 0.001/****P < 0.0001 compared to 0.5 (one sample t-test) for the NOR, and *P < 0.05/**P < 0.01/***P < 0.001/****P < 0.0001 compared to sham-operated WT or APP/PS1 mice (two-tailed unpaired t-test) for the remaining analysis.

Some observations indicated that the presence of microinfarcts in young AD patients is associated with worsening of the clinical outcomes. The impact of microinfarct occurrence at the early stages of AD on cognitive decline remains elusive. As in APP/PS1 mice the cognitive decline is progressive, the consequences of microinfarct induction of cognitive functions were evaluated using different neurobehavioral paradigms to assess recognition memory using the NOR test (**Figure 3A**) and to examine willingness to explore and anxiety-like behavior using the OF test (**Figure 3B**). Analysis of NOR discrimination index showed that 1 week after microinfarct induction, MO-operated male WT mice and APP/PS1 mice exhibited an impaired recognition memory in comparison to sham-operated mice (**Figure 3C**), an effect that was maintained for 1 month in sham-operated APP/PS1 mice in which AD-like pathology normally progressed, and MO-operated WT mice and APP/PS1 mice (**Figure 3D**). Furthermore, 1 week after microinfarct induction, NOR discrimination analysis indicated that, similarly to males, the recognition memory was impaired in MO-operated female WT mice and APP/PS1 mice in comparison to sham-operated mice (**Figure 3E**). Interestingly, 1 month after microinfarct induction, the recognition memory was recovered in MO-operated female WT mice and APP/PS1 mice, as well as sham-operated WT mice, except in sham-operated APP/PS1 mice in which AD-like pathology normally progressed (**Figure 3F**). The OF test analysis indicated that the time spent in the center was high in MO-operated male APP/PS1 mice in comparison to sham-operated mice, indicative of a reduced state of vigilance translated by an increased disinhibitory behavior, whereas no differences were observed between sham- and MO-operated WT mice (**Figure 3G**). In parallel, the distance travelled by MO-operated WT mice and APP/PS1 mice significantly increased in comparison to sham-operated mice, with an exaggerated effect in young APP/PS1 mice (**Figure 3H**), outlining a reduced state of vigilance as well. Interestingly, the patterns of the time spent in the center (**Figure 3I**) and the travelled distance (**Figure 3J**) were maintained in MO-operated WT mice and APP/PS1 mice 1 month later, indicating a longitudinal preserved effect of microinfarcts. On the other hand, the time spent in the center remained unchanged among sham- and MO-operated female WT mice and APP/PS1 mice (**Figure 3K**). The distance travelled was increased specifically in MO-operated female WT mice, whereas it remained unchanged in MO-operated female APP/PS1 mice (**Figure 3L**). In contrast to young males, the time spent in the center remained unchanged 1 month later in MO-operated female WT mice and APP/PS1 mice in comparison to sham-operated mice (**Figure 3M**). Furthermore, the distance travelled by MO-operated female WT mice and APP/PS1 mice increased in comparison to sham-operated littermates, with female APP/PS1 mice showing a moderate increase (**Figure 3N**). Our findings suggest that microinfarcts caused a rapid and prolonged memory impairment in young male APP/PS1 mice, accompanied by a reduced state of vigilance and an increased disinhibitory behavior, independently upon AD-like pathology, whereas in female APP/PS1 mice, these effects were mild and transient.

### Multifocal Microinfarcts Trigger Prolonged Functional and Structural Vascular Deregulations

**Figure 4:**
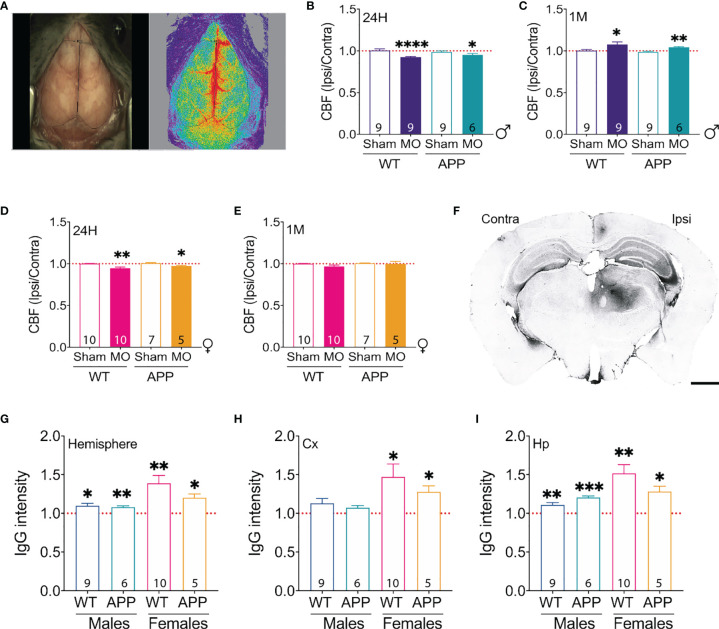
Induction of multifocal microinfarcts is associated with prolonged cerebrovascular dysfunctions. **(A)** Representative images of the LCSI system used to investigate the CBF. Ratio of the CBF in the ipsilateral hemisphere over the contralateral hemisphere was computed and analyzed. LCSI analysis shows that the CBF **(B)** decreases in the ipsilateral hemisphere 24 hours post-microinfarct induction, while **(C)** it increases 1 month later in MO-operated male WT and MO-operated male APP/PS1. In females, LCSI analysis shows that the CBF **(D)** decreases in the ipsilateral hemisphere 24 hours post-microinfarct induction, and **(E)** is restored 1 month later in MO-operated female WT and MO-operated female APP/PS1. A representative image of **(F)** IgG immunolabeling indicative of extravasation in the brain of MO-operated male WT mice. Analysis of IgG immunolabeling in the overall hemisphere **(G)** indicates that IgG infiltration is increased in the ipsilateral hemisphere of MO-operated male and female WT as well as APP/PS1 mice. Analysis of IgG specific immunolabeling in the cortex **(H)** indicates that IgG infiltration remains unchanged among MO-operated male APP/PS1 mice and MO-operated male WT mice, whereas it similarly increases in the ipsilateral cortex of MO-operated female WT mice and MO-operated female WT mice. Analysis of IgG immunolabeling in the hippocampus **(I)** indicates that IgG infiltration increases in the ipsilateral hippocampus of MO-operated male and female WT as well as APP/PS1 mice. Data are mean ± SEM (n = 5-10 animals/group). *P < 0.05/**P < 0.01/****P < 0.0001 compared to 1 (one sample t-test) for the CBF and IgG ratios. Scale bar = 1000 µm.

Although the impact of CBF deregulation and vascular permeability have been shown to constitute an important risk factor for AD (6, 63, 64), little is known about how these parameters are impacted following the occurrence of microinfarcts. Using LCSI to assess the regional changes in relative CBF (**Figure 4A**), our analysis showed that the overall CBF value in the ipsilateral hemisphere is reduced 24 hours after microinfarct induction in MO-operated male WT mice and APP/PS1 mice (**Figure 4B**), indicating an acute impairment of neurovascular coupling associated with cerebral hypoperfusion. Interestingly, 1 month later, the absolute relative CBF value in the ipsilateral hemisphere of MO-operated male WT mice and APP/PS1 mice increased beyond those of the contralateral hemisphere (**Figure 4C**), outlining a possible dysfunction of the autoregulation process over time associated with cerebral hyperperfusion. Like males, LCSI analysis indicated that the CBF in the ipsilateral hemisphere is reduced 24 hours after microinfarct induction in MO-operated female WT mice and APP/PS1 mice (**Figure 4D**). However, we found that the CBF was completely recovered in the ipsilateral hemisphere of MO-operated female WT mice and APP/PS1 mice after 1 month (**Figure 4E**). Next, the impact of microinfarcts on BBB permeability was assessed 1 month later using IgG immunohistology (**Figure 4F**). Our analysis showed that overall IgG infiltration into the ipsilateral hemisphere increased in MO-operated WT mice and APP/PS1 mice independently upon the biological sex (**Figure 4G**), outlining the presence of permeable BBB at this stage. Interestingly, IgG infiltration in the ipsilateral cortex was increased specifically in MO-operated females and to a lesser extent in MO-operated males, thus highlighting a more potent impact vascular permeability in the brain of female compared to male mice (**Figure 4H**). Finally, we found that IgG infiltration into the ipsilateral hippocampus was more potent in MO-operated female APP/PS1 mice (**Figure 4I**). Our results indicate that the microinfarcts caused a prolonged CBF deregulation in the ipsilateral hemisphere of male APP/PS1 mice, independently upon AD-like pathology, whereas the CBF alterations in the ipsilateral hemisphere of female APP/PS1 mice were acute and associated to permeable BBB.

### The Mechanisms Associated With Aβ Turnover Remain Unaffected Upon Microinfarction

**Figure 5:**
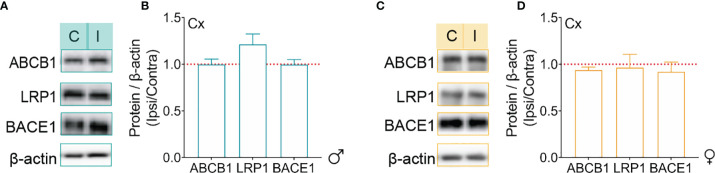
Mechanisms of Aβ production and clearance are not affected by microinfarct induction. **(A)** Western blot representation of ABCB1, LRP1 and BAEC1 in the contralateral and ipsilateral cortex of MO-operated male APP/PS1 mice. **(B)** Western blot analysis of ipsilateral and contralateral expression ratio shows that there are no changes in ABCB1, LRP1 and BACE1 expression in the cortex of MO-operated male APP/PS1 mice. **(C)** Western blot representation of ABCB1, LRP1 and BAEC1 in the contralateral and ipsilateral and cortex of MO-operated female APP/PS1 mice. **(D)** Western blot analysis of ipsilateral and contralateral expression ratio indicates that the expression of ABCB1, LRP1 and BACE1 remains unchanged in the cortex of MO-operated female APP/PS1 mice. Statistical analysis was performed by comparing the ratios of ipsilateral/contralateral to 1 (one sample t-test).

It has been previously shown that several mechanisms associated with Aβ clearance and production are impaired in AD (35, 52). As such, the effects of microinfarcts on the expression of some key markers, including ABCB1 and LRP1, which are both involved in facilitating Aβ elimination from the brain to the periphery across the BBB (65), as well as BACE1, which plays a key role in generating Aβ peptides *via* the cleavage of APP (66, 67), was evaluated using Western blot analysis. Our data indicated that the protein levels of ABCB1, LRP1, and BACE1 remained unchanged in the ipsilateral hemisphere of MO-operated male (**Figures 5A, B**) as well as female (**Figures 5C, D**) WT mice and APP/PS1 mice 1 month after microinfarct induction. Our results suggest that the mechanisms implicated in Aβ vascular elimination or parenchymal production are not affected by the microinfarcts 1 month later.

### Microglia Are Activated After Multifocal Microinfarcts Dependently Upon Biological Sex

**Figure 6:**
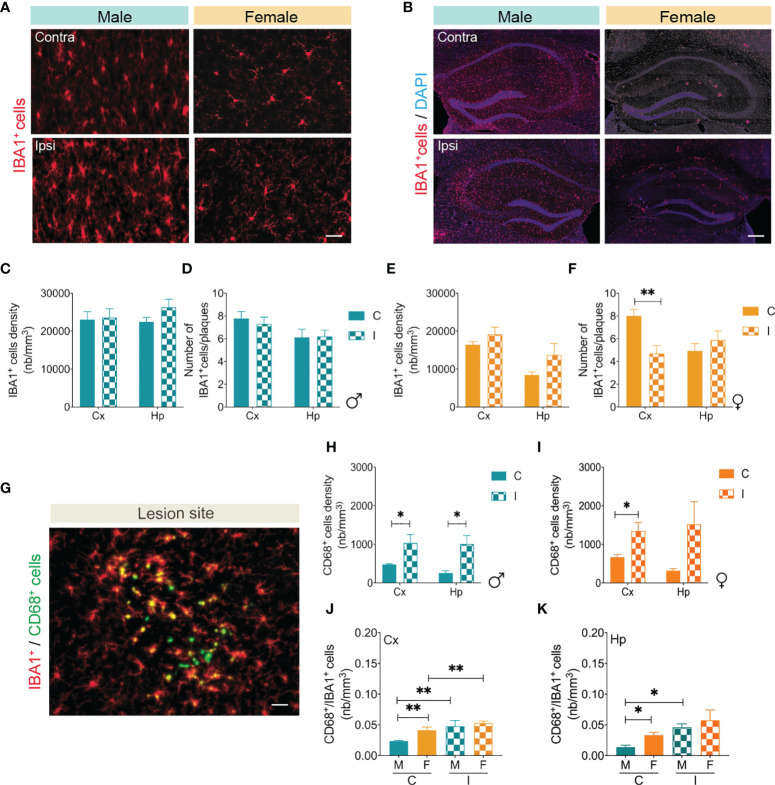
Multifocal microinfarcts induce microglial cell activation. Representative fluorescent images of IBA1 immunolabeled microglia (red) in the **(A)** cortex and **(B)** hippocampus of MO-operated male and female APP/PS1 mice. Immunofluorescence combined to stereological analysis shows that **(C)** the density of IBA1^+^ cells in the cortex and hippocampus, and **(D)** the number of IBA1^+^ cells surrounding Aβ plaques remains unchanged in the brain of MO-operated male APP/PS1 mice. Immunofluorescence combined to stereological analysis shows that **(E)** the density of IBA1^+^ cells in the cortex and hippocampus of MO-operated male APP/PS1 mice remains unchanged, and while **(F)** the number of IBA1^+^ cells surrounding Aβ plaques decreases in the cortex of MO-operated female APP/PS1 mice, it remains unchanged in the hippocampus. **(G)** A representative fluorescent image of IBA1 immunolabeled microglia (red) and CD68 (green) at the lesion site in MO-operated male WT mice show that microglia adopt a phagocytic state (IBA1^+^/CD68^+^). A population of IBA1^-^/CD68^+^ cells phagocytic cells are also present. Immunofluorescence combined to stereological analysis shows that the density of CD68^+^ cells in the ipsilateral hemisphere of **(H)** MO-operated male APP/PS1 mice, and **(I)** MO-operated female APP/PS1 mice. Immunofluorescence combined to stereological analysis indicates that **(J)** the density of IBA1^+^/CD68^+^ cells is higher in the contralateral cortex of MO-operated female APP/PS1 mice compared to the contralateral cortex of MO-operated male APP/PS1 mice and remains higher in the ipsilateral cortex of MO-operated female compared to the ipsilateral cortex of MO-operated male APP/PS1 mice. Immunofluorescence combined to stereological analysis indicates that **(K)** the density of IBA1^+^/CD68^+^ cells is increased in the contralateral hippocampus of MO-operated female APP/PS1 mice compared to MO-operated male APP/PS1 mice, as well as in the ipsilateral hippocampus of MO-operated male APP/PS1 mice compared to the contralateral hippocampus of MO-operated male APP/PS1 mice. Data are mean ± SEM (n = 5-10 animals/group). *P < 0.05/**P < 0.01 compared to ipsilateral or contralateral hemisphere of MO-operated male and female APP/PS1 mice (two-tailed unpaired t-test). Scale bar = 250 µm.

At the early stages of AD, microglial cell activation slows disease progression *via* elimination of Aβ through phagocytosis (65, 68). Reports have indicated that microglial cells are activated in response to microinfarcts within the lesion sites (55). Moreover, microglial cell activation has been proposed to be influenced by biological sex, and that microglia isolated from females adopt a neuroprotective, phagocytic phenotype in response to ischemic challenge (69). Using immunohistochemical analysis (**Figures 6A, B**), we investigated the impact of multifocal microinfarct induction on microglial cell behavior in the brain of male and female APP/PS1 mice. Our immunofluorescence analysis shows that microglial cell overall density (IBA1 immunolabeled) remained unchanged in the cortex and hippocampus of MO-operated male APP/PS1 mice 1 month after induction of multifocal cerebral microinfarcts (**Figure 6C**). It has been previously shown that microglia are recruited to Aβ plaques (18, 65, 70). Our colocalization investigations indicated that the number of microglial cells (IBA1) surrounding Aβ plaques (6E10) was not affected 1 month after the induction of microinfarcts in MO-operated male APP/PS1 mice (**Figure 6D**). Similarly, the overall density of microglia remained unchanged in the cortex and hippocampus of MO-operated female APP/PS1 mice (**Figure 6E**). However, we observed a specific reduction in the number of microglial cells surrounding Aβ plaques in the cortex of MO-operated female APP/PS1 mice (**Figure 6F**). Next, microglial cell relative phagocytic capacity was evaluated by assessing expression of the lysosomal receptor CD68 (**Figure 6G**). Our analysis shows that CD68 expression increased in the ipsilateral cortex and hippocampus of MO-operated male (**Figure 6H**) and female (**Figure 6I**) APP/PS1 mice. Furthermore, the number of IBA1-immunolabeled cells expressing CD68 increased in the cortex (**Figure 6J**) and hippocampus (**Figure 6K**) of MO-operated APP/PS1 mice, exhibiting similar colocalization patterns between males and females. Our findings indicate that the microinfarcts triggered microglial activation and phagocytic capabilities similarly in the brain of male and female APP/PS1 mice, which may account for the attenuated Aβ pathology.

### Frequency of Circulating Monocytes Is Regulated in Response to Multifocal Microinfarcts

**Figure 7:**
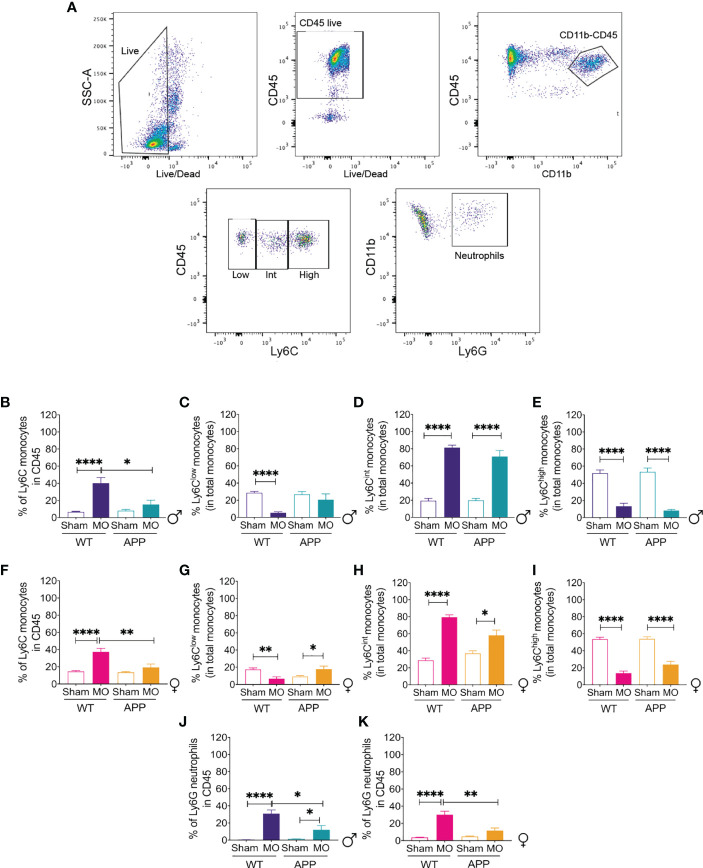
Dynamics of circulating monocytes are modulated upon multifocal microinfarction. **(A)** Gating strategy used to discriminate the circulating monocytes (CD11b^+^ LyC6^+^) in leukocytes (CD45^+^) and the subsequent distribution of the inflammatory monocytes (Ly6C^high^), patrolling monocytes (Ly6C^low^) and neutrophils (Ly6G^high^). **(B)** Flow cytometry analysis shows that the frequency of total monocytes in the blood circulation increases in MO-operated male WT mice while it remains unchanged in MO-operated male APP/PS1 mice compared to sham-operated male mice and decreases in MO-operated male APP/PS1 mice compared to MO-operated male WT mice. **(C)** Flow cytometry analysis shows that the frequency of Ly6C^low^ monocytes decreases in the blood circulation of MO-operated male WT mice compared to sham-operated WT mice. **(D)** Flow cytometry analysis shows that the frequency of Ly6C^int^ monocytes increases in the blood circulation of MO-operated male WT mice as well as MO-operated male APP/PS1 mice compared to sham-operated WT mice. **(E)** Flow cytometry analysis shows that the frequency of inflammatory Ly6C^high^ monocytes decreases in the blood circulation of MO-operated male WT mice as well as MO-operated male APP/PS1 mice compared to sham-operated male mice. **(F)** Flow cytometry analysis shows that similarly to males, the frequency of total monocytes in the blood circulation increases in MO-operated female WT mice while it remains unchanged in MO-operated female APP/PS1 mice compared to sham-operated female mice and decreases in MO-operated female APP/PS1 mice compared to MO-operated female WT mice. **(G)** Flow cytometry analysis indicates that the frequency of Ly6C^low^ monocytes increases in MO-operated female WT mice, while it decreases in the MO-operated female APP/PS1 mice compared to sham-operated female mice. **(H)** Flow cytometry analysis indicates that the frequency of Ly6C^int^ monocytes increases in the blood circulation of MO-operated female WT mice and to a lesser extent MO-operated female APP/PS1 mice. **(I)** Flow cytometry analysis indicates that the frequency of Ly6C^high^ monocytes is decreased in the blood circulation of MO-operated female WT mice as well as MO-operated female APP/PS1 mice compared to sham-operated female mice. **(J)** Flow cytometry analysis shows that the frequency of Ly6G neutrophils increases in MO-operated male WT mice compared to sham-operated male WT mice but to a lesser extent in MO-operated male APP/PS1 mice compared to sham-operated APP/PS1 male mice. **(K)** Flow cytometry analysis shows that the frequency of Ly6G neutrophils similarly increases in the blood circulation of MO-operated female WT mice compared to sham-operated female WT mice, whereas it remains unchanged in MO-operated female APP/PS1 mice compared to sham-operated female APP/PS1 mice. Data are mean ± SEM (n = 5-10 animals/group). *P < 0.05/**P < 0.01/****P < 0.01 compared with sham- and MO-operated male and female WT and APP/PS1 mice (two-tailed unpaired t-test).

Monocytes play an important role in AD (19, 71). The inflammatory monocyte subset infiltrates the brain and differentiates into microglia-like cells that contribute to Aβ clearance *via* phagocytosis (14, 71). The patrolling monocyte subset contributes to the clearance of Aβ vascular micro-aggregates (18). A progressive defective production of Ly6C^low^ CX3CR1^high^ and Ly6C^high^ CX3CR1^low^ monocyte subsets, coinciding with cognitive decline, has been reported in APP/PS1 mice (71). Using flow cytometry analysis, we analyzed the response of the different monocyte subsets and neutrophils (**Figure 7A**) to multifocal microinfarcts in male and female WT mice and APP/PS1 mice. Our analysis showed that 48 hours after microinfarct induction, an increased frequency of total monocytes was observed in the blood circulation of MO-operated male WT mice, which was absent in MO-operated male APP/PS1 mice (**Figure 7B**). Nonetheless, monocyte frequency in MO-operated male APP/PS1 mice was significantly lower in comparison to MO-operated male WT mice (**Figure 7B**). Analysis of the different monocyte populations showed a reduced frequency of Ly6C^low^ monocytes in the blood circulation of MO-operated male WT mice in comparison to sham-operated mice, whereas no changes were observed in sham- and MO-operated male APP/PS1 mice (**Figure 7C**). The frequency of Ly6C^int^ monocytes significantly increased in the blood circulation of MO-operated male WT mice and APP/PS1 mice in comparison to sham-operated mice (**Figure 7D**), associated with a decreased frequency of Ly6C^high^ monocytes (**Figure 7E**). Additionally, MO-operated female WT exhibited higher total monocyte frequency in the blood circulation in comparison to female MO-operated APP/PS1 mice, which was absent in MO-operated female APP/PS1 mice (**Figure 7F**). Interestingly, monocyte frequency in MO-operated female APP/PS1 mice was reduced to a lesser extent in comparison to MO-operated female WT mice (**Figure 7F**). Moreover, the frequency of Ly6C^low^ monocytes was elevated in the blood circulation of MO-operated female WT mice in comparison to sham-operated mice, whereas in contrast to males, the frequency of Ly6C^low^ monocytes increased in the blood circulation of female MO-operated APP/PS1 mice (**Figure 7G**). Similar to males, the frequency of Ly6C^int^ monocytes increased in the blood circulation of MO-operated female WT mice and to a lesser extent of MO-operated female APP/PS1 mice in comparison to sham-operated mice (**Figure 7H**), accompanied with a decreased frequency of Ly6C^high^ monocytes (**Figure 7I**). Finally, the frequency of Ly6G^high^ neutrophils was increased in the blood circulation of MO-operated male WT, but to a lesser extent in MO-operated APP/PS1 mice (**Figure 7J**). Similarly, MO-operated female WT mice exhibited an increased frequency in circulating neutrophils, which was absent in MO-operated female APP/PS1 mice (**Figure 7K**). Moreover, MO-operated female APP/PS1 mice had a lower frequency of neutrophils in the blood circulation compared to the MO-operated WT females (**Figure 7K**). Our results suggest that microinfarcts stimulated the generation of Ly6C^int^ monocytes and induced a specific increase in Ly6C^low^ CX3CR1^high^ monocytes in the blood circulation of female APP/PS1 mice.

### Peripheral Phagocytic Cells Infiltrate the Brain Following Multifocal Microinfarct Induction

**Figure 8:**
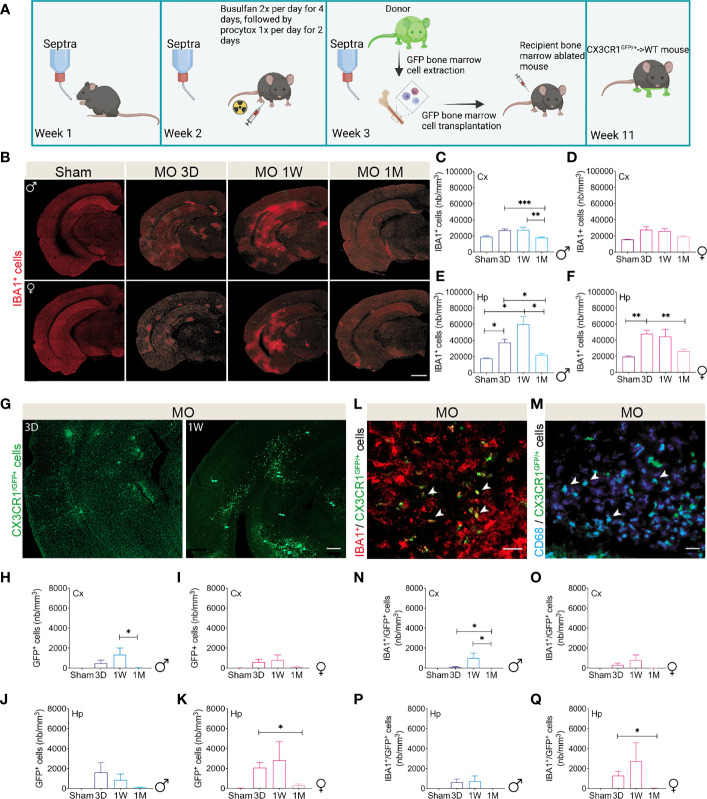
Multifocal microinfarcts stimulate the infiltration of peripheral phagocytic cells into the brain. **(A)** A scheme illustrating the strategy used to generate CX3CR1^GFP/+^ → WT chimeric mice. WT mice were subjected to a chemotherapy regimen to ablate bone marrow-derived myeloid cells and transplanted with bone marrow cells from donor CX3CR1^GFP/+^ mice in which Ly6C^low^ CX3CR1^high^ cells are GFP^+^. Created with BioRender.com. **(B)** Representative fluorescent images of IBA1 immunolabeled microglia (red) in the brain of MO-operated male and female WT mice at 3 days, 1 week and 1month post-microinfarct induction. **(C)** Immunofluorescence combined to stereological analysis indicates that the density of IBA1^+^ cells, increases in the cortex of MO-operated male CX3CR1^GFP/+^ → WT chimeric mice, peaking at 3 days, and remaining elevated until 1 week, and then decreases 1 month post-microinfarct induction. **(D)** Immunofluorescence combined to stereological analysis shows a similar trend in the cortex of MO-operated female CX3CR1^GFP/+^ → WT chimeric mice without reaching a statistical significance. **(E)** Immunofluorescence combined to stereological analysis indicates that the density of IBA1^+^ cells, increases in the hippocampus of MO-operated male CX3CR1^GFP/+^ → WT chimeric mice, peaking at 3 days, and remaining elevated until 1 week, followed by a decrease 1 month post-microinfarct induction. **(F)** Immunofluorescence combined to stereological analysis shows a similar trend in the hippocampus of MO-operated female CX3CR1^GFP/+^ → WT chimeric mice. Representative fluorescent images show that CX3CR1^GFP/+^ cells are massively recruited into the brain of MO-operated male CX3CR1^GFP/+^ → WT chimeric mice, **(G)** 3 days and 1 week post-microinfarct induction. Immunofluorescence combined to stereological analysis indicates that the number of CX3CR1^GFP/+^ cells peaks in the cortex of **(H)** MO-operated male CX3CR1^GFP/+^ → WT chimeric mice, and to a lesser extent in **(I)** females, 1 week post-microinfarct induction. **(J)** Immunofluorescence combined to stereological analysis indicates that the number of CX3CR1^GFP/+^ cells in the hippocampus of MO-operated male CX3CR1^GFP/+^ → WT chimeric mice progressively increases at 3 days and decreases 1 month post-microinfarct induction. **(K)** Immunofluorescence combined to stereological analysis indicates that the number of CX3CR1^GFP/+^ cells in the hippocampus of MO-operated female CX3CR1^GFP/+^ → WT chimeric mice increases 1 week and decreases 1 month post-microinfarct induction. **(L)** A representative fluorescent image showing the colocalization of microglia (IBA1-immunolabeled) and bone marrow-derived CX3CR1^GFP/+^ cells at the lesion site. **(M)** A representative fluorescent image showing expression of the phagocytic maker CD68 in bone marrow derived CX3CR1^GFP/+^ cells at the lesion site. **(N)** Immunofluorescence combined to stereological analysis indicates that the density of IBA1^+^/CX3CR1^GFP/+^ cells increases the cortex of MO-operated male CX3CR1^GFP/+^ → WT chimeric mice 1 week and decreases 1 month post-microinfarct induction. **(O)** Immunofluorescence combined to stereological analysis outlines a similar trend in MO-operated female CX3CR1^GFP/+^ → WT chimeric mice without reaching statistical significance. Immunofluorescence combined to stereological analysis indicates that the density of IBA1^+^/CX3CR1^GFP/+^ cells in the hippocampus of **(P)** MO-operated male CX3CR1^GFP/+^ → WT chimeric mice remains unchanged, while **(Q)** it increases in females 1 week post-microinfarct induction. Data are mean ± SEM (n = 5-10 animals/group). *P < 0.05/*P < 0.01/***P < 0.001 compared to sham- and MO-operated male and female CX3CR1^GFP/+^ → WT chimeric mice at different time points (two-tailed unpaired t-test). Scale bar = 500 µm **(B)** and 150 µm **(G, L, M)**.

In addition to resident activated microglia, CD68 is highly expressed as well in peripheral mononuclear phagocytes, namely CX3CR1^high^ monocytes (25, 72). Moreover, Ly6C^low^ CX3CR1^high^ monocytes have been previously shown to transiently infiltrate the brain to promote neuroprotection in response to excitotoxicity (72). As our findings above suggest that not all CD68-reactive cells are resident microglia, we were interested in investigating the brain dynamics of Ly6C^low^ CX3CR1^high^ monocytes that differentially respond to cerebral microinfarction. For this purpose, chimeric mice enabling the specific tracking of Ly6C^low^ CX3CR1^high^ monocytes, were generated in male and female WT mice, which were chosen to eliminate any confounding effects associated with Aβ pathology. This was achieved by subjecting male and female WT mice to a chemotherapy regimen to ablate the bone marrow-derived myeloid cells followed by the transplantation of bone marrow-derived cells isolated from respectively male and female CX3CR1^GFP/+^ mice (CX3CR1^GFP/+^ → WT chimeric mice) (**Figure 8A**), as previously described (18). Flow cytometry analysis indicated that the reconstituted bone marrow cells comprised 90% ± 1.78% of CX3CR1^GFP/+^ monocytes. As IBA1 could be expressed as well in infiltrating macrophages, its reactivity in the brain of male and female CX3CR1^GFP/+^ → WT chimeric mice was investigated at different time points representing the acute (3 days), sub-acute (1 week) and chronic (1 month) phases after microinfarct induction (**Figure 8B**). Our immunofluorescence analysis shows that the number of IBA1^+^ cells increased beginning of day 3, peaking at 1 week, and returning to the basal levels 1 month later in the ipsilateral cortex of MO-operated male (**Figure 8C**) and female (**Figure 8D**) CX3CR1^GFP/+^ → WT chimeric mice, thus exhibiting temporal transient increase independently upon biological sex. Similar IBA1 reactivity patterns were observed in the hippocampus of MO-operated male (**Figure 8E**) and female (**Figure 8F**) CX3CR1^GFP/+^ → WT chimeric mice. We next assessed the infiltration of Ly6C^low^ CX3CR1^high^ monocytes into the brain of MO-operated CX3CR1^GFP/+^ → WT chimeric mice, and we found out that CX3CR1^GFP/+^ cells were massively recruited to the ipsilateral brain between day 3 and week 1, whereas they were not detectable in the ipsilateral brain 1 month later (**Figure 8G**). Stereological analysis indicated that GFP^+^ cells were detected at day 3, peaked at week 1, and were almost absent in the ipsilateral cortex of MO-operated male (**Figure 8H**) as well as female (**Figure 8I**) CX3CR1^GFP/+^ → WT chimeric mice 1 month later. However, in the ipsilateral hippocampus, our analysis showed that the density of GFP^+^ cells was higher at day 3 and begun to decrease from week 1 until 1 month later in MO-operated male CX3CR1^GFP/+^ → WT chimeric mice (**Figure 8J**), whereas in MO-operated females, GFP^+^ cell density was higher at 1 week (**Figure 8K**). Finally, some infiltrated GFP^+^ cells (i.e. CX3CR1 monocytes) were found to express IBA1 (**Figure 8L**), as well as and CD68 (**Figure 8M**), outlining their potential phagocytic capacity. Analysis of GFP^+^ and IBA1^+^ colocalization shows that the density of GFP^+^ cells expressing IBA1 peaked at 1 week in the ipsilateral cortex of MO-operated male (**Figure 8N**) and female (**Figure 8O**) CX3CR1^GFP/+^ → WT chimeric mice. Interestingly, although similar colocalization pattern was observed in the ipsilateral hippocampus of MO-operated male CX3CR1^GFP/+^ → WT chimeric mice (**Figure 8P**), higher density of GFP^+^ cells expressing IBA1 was observed in females (**Figure 8Q**). Our findings suggest that the multifocal cerebral microinfarcts promoted the infiltration of circulating Ly6C^low^ CX3CR1^high^ monocytes that exhibited a phagocytic potential more potently in the hippocampus of female mice.

### Infiltrated Phagocytic Monocytes Are Actively Recruited to the Lesion Sites and Aβ Plaques

**Figure 9:**
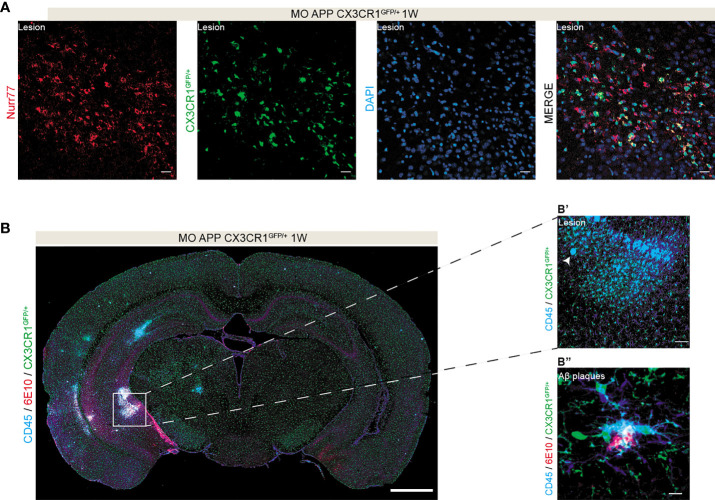
Infiltrated phagocytic monocytes are recruited to the lesion sites and Aβ plaques. **(A)** A tile representing a coronal brain section of MO-operated male APP/PS1/CX3CR1^GFP/+^ mouse immunolabeled with CD45 (i.e. infiltrating cells, blue) and IBA1 (i.e. microglia; red) showing **(A)** the presence of CX3CR1^GFP/+^ expressing Nurr77^+^, highlighting their monocytic origin, at lesion site 1 week post-microinfarct induction. **(B)** A tile representing a coronal brain section of MO-operated male APP/PS1/CX3CR1^GFP/+^ mouse immunolabeled with CD45 (blue) and 6E10 (Aβ plaques; red) showing **(B’)** the presence of CD45^+^/CX3CR1^GFP/+^ cells at the lesion site and **(B’’)** CD45^+^/IBA1^+^/CX3CR1^GFP/+^ cells recruited to Aβ plaques. Scale bar = 20 µm **(A)**, 1000 µm **(B)**, 50 µm **(B’)** and 25 µm **(B”)**.

As Ly6C^low^ CX3CR1^high^ monocytes were shown here to efficiently infiltrate the brain after multifocal microinfarcts in absence of Aβ pathology, we investigated next the behavior of infiltrated cells in the brain of APP/PS1 mice. For this purpose, we used APP/PS1/CX3CR1^GFP/+^ triple transgenic mice in which Ly6C^low^ CX3CR1^high^ monocytes as well as resident microglia are GFP^+^. Brain infiltrated phagocytic Ly6C^low^ CX3CR1^high^ monocytes were discriminated from resident microglia through their expression of Nurr77, a transcription factor implicated in the survival of bone marrow derived Ly6C^low^ monocytes (18). Our immunofluorescence analysis indicates that GFP^+^ cells expressing Nurr77 (i.e. CX3CR1 monocytes) are specifically recruited to the lesion site (**Figure 9A**) in the brain MO-operated male APP/PS1/CX3CR1^GFP/+^ mice 1 week after microinfarct induction. Interestingly, our analysis suggests that CX3CR1^GFP/+^ cells expressing CD45 (i.e. infiltrating immune cells) co-localized with Aβ plaques immunolabeled with 6E10 (**Figure 9B**). As no major differences were observed in the infiltration rate of Ly6C^low^ CX3CR1^high^ monocytes in the brains of males and females, only males were used for this experiment. Our results suggest that the infiltrated CX3CR1^high^ monocytes are possibly contributing to the remodeling of injured tissue and to Aβ clearance, which may account for the attenuated Aβ pathology.

### Induction of Multifocal Microinfarcts Triggers DKK1 Expression in a Sex-Specific Manner

**Figure 10:**
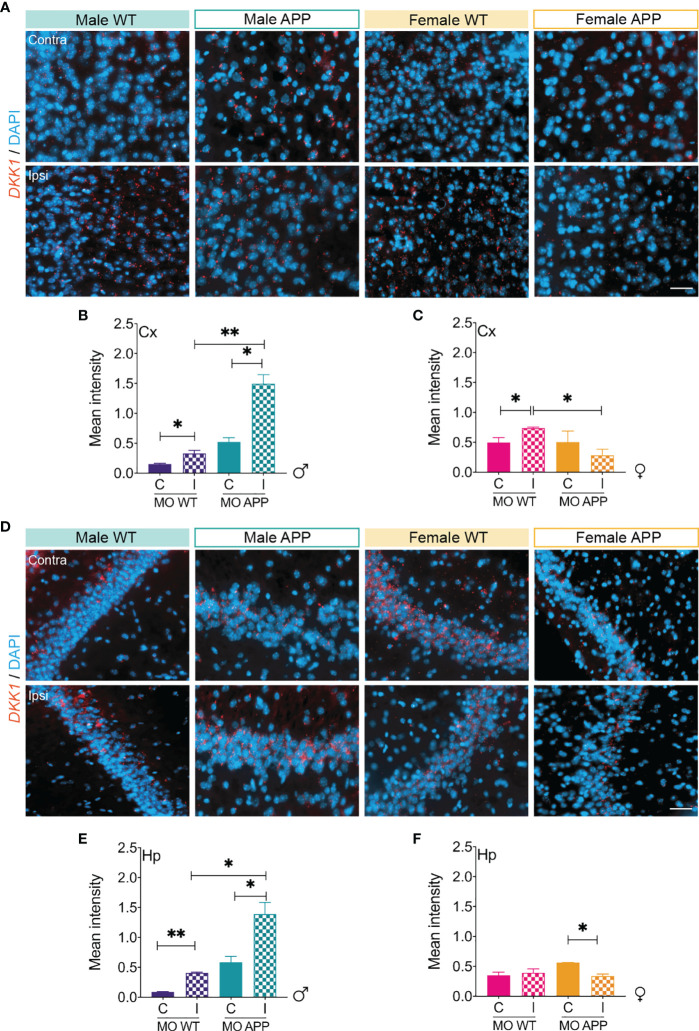
DKK1 expression is increased in response to microinfarction dependently upon biological sex. **(A)** Representative images of FISH assay showing *DKK1* mRNA transcripts (red) and DAPI (blue) in the contralateral and ipsilateral cortex of MO-operated male and female WT and APP/PS1 mice. **(B)** Analysis of the fluorescence intensity indicates that the expression of *DKK1* mRNA is slightly increased in the ipsilateral cortex of MO-operated male WT mice, which is more pronounced in the ipsilateral cortex of MO-operated male APP/PS1 mice. **(C)** Analysis of the fluorescence intensity indicates that the expression of *DKK1* mRNA is slightly increased in the ipsilateral cortex of MO-operated female WT mice, while it is deceased in the ipsilateral cortex of MO-operated APP/PS1 mice compared to MO-operated WT animals. **(D)** Representative images of FISH assay showing *DKK1* mRNA transcripts (red) and DAPI (blue) in the contralateral and ipsilateral hippocampus of MO-operated male and female WT and APP/PS1 mice. **(E)** Analysis of the fluorescence intensity indicates that the expression of *DKK1* mRNA is slightly increased in the ipsilateral cortex of MO-operated male WT mice, which similarly to the cortex is more pronounced in the ipsilateral cortex of MO-operated male APP/PS1 mice. **(F)** Analysis of the fluorescence intensity indicates that the expression of *DKK1* mRNA remains unchanged in the ipsilateral hippocampus of MO-operated female WT mice, while it tends to decrease in the hippocampus of MO-operated female APP/PS1 mice. Data are mean ± SEM (n = 5-10 animals/group). C, Contralateral; I, Ipsilateral. *P < 0.05/**P < 0.01 compared with sham- and MO-operated WT or APP/PS1 mice (two-tailed unpaired t-test). Scale bar = 50 µm.

As the changes reported here in Aβ pathology do not correlate with cognitive decline following the induction of microinfarcts, we were interested in identifying the possible underlying mechanisms. DKK1 has been shown to be induced by ischemic/hypoxic insults (73), to be implicated in AD pathogenesis (52), and to mediate cognitive decline independently of Aβ pathology *via* inhibition of synaptic transmission (74). For this purpose, the expression of DKK1, an endogenous inhibitor of the canonical Wnt pathway was evaluated in the cortex of mice 1 month after microinfarct induction using FISH RNAscope assay (**Figure 10A**). Our analysis indicated that intensity *DKK1* mRNA transcripts increased in the ipsilateral cortex of MO-operated male WT mice, which was exacerbated in MO-operated male APP/PS1 mice (**Figure 10B**). Interestingly, *DKK1* mRNA levels remained low in the cortex of MO-operated female APP/PS1 mice (**Figure 10C**). We next investigate the expression pattern of DKK1 mRNA in the hippocampus 1 month after microinfarct induction (**Figure 10D**) and found out that *DKK1* mRNA levels increased in the ipsilateral hippocampus of MO-operated male WT mice, which was exacerbated as well in MO-operated male APP/PS1 mice (**Figure 10E**). Finally, we found out that *DKK1* mRNA levels remained unchanged in the ipsilateral hippocampus of MO-operated female WT mice, and significantly decreased in the hippocampus of MO-operated female APP/PS1 mice (**Figure 10F**). Our findings suggest that DKK1 expression is potently induced in the brain of male APP/PS1 mice after microinfarcts, which may account for the exaggerated cognitive decline in males.

## Discussion

Major efforts have been invested in the past decade to understand the role of vascular risk factors in AD etiology, pathogenesis and progression (9–11). The overwhelming observations clearly outlined an increased risk of cerebral microinfarct incidence associated with vascular pathologies in AD patients (26–30). Although AD affects more women than men in the elderly population, young women are protected against developing AD as well as cerebrovascular diseases, which is thought to be associated with the protective effects of female sexual hormones, namely estrogens (47–51). However, this advantage seems to be lost and reversed with age, and more particularly with menopause in females (75, 76). Despite these observations, the impact of multifocal cerebral microinfarcts that occur at the early stages of AD on disease pathobiology in males and females remains not fully elucidated. In this study, we investigated the consequences of sporadic microinfarcts induced *via* the occlusion of the cerebral microvasculature on AD-like pathology in young male and female APP/PS1 mice, a widely used model characterized by progressive Aβ pathology and cognitive decline. Multifocal microinfarcts were induced using a novel approach that was recently developed to induce CSVD in mice *via* the occlusion of penetrating arteries and capillaries using micro-emboli (55). The age of animals was carefully chosen to reflect the early stages of AD-like pathology in APP/PS1 mice to better appreciate the impact of microinfarcts, which can be overwhelmed by the advanced AD-like pathology in older mice. Interestingly, we noted first a sex-dependent effect on Aβ deposition in the brain of young APP/PS1 mice. For instance, the overall density of Aβ plaques was almost 1.5 times more important in females in comparison to males, whereas the mean volume of individual Aβ plaques was 2 times higher in males in comparison to females. However, there were no major differences in the cognitive deficits at this stage, suggesting that Aβ peptides aggregated differently in males and females. Our results are in line with previous reports indicating that AD neuropathology is indeed different between males and females in humans as well as in animal models (77–80). These reports outlined a more progressive AD pathology in aged females in comparison to males. Nonetheless, our study provided for the first-time evidence that Aβ deposition is fundamentally different in young male and female APP/PS1 mice, with females having more Aβ plaques but of smaller volume, an important aspect that should be taken into consideration when investigating Aβ pathology in APP/PS1 mice. Afterwards, we found out that the early induction of multifocal microinfarcts unexpectedly reduced the density of Aβ plaques in the brain APP/PS1 females and the volume of individual Aβ plaques in the brain APP/PS1 males. Our results suggest that microinfarcts reduced overall Aβ plaque deposition in the brain of APP/PS1 mice in respect to the specificity of biological sex. It has been shown that the burden of Aβ plaques in the brain does not necessarily correlate with the cognitive decline observed in AD, whereas the detrimental role of soluble Aβ oligomers on synaptic function and plasticity is well documented (81, 82). We found out in our study that the levels of soluble Aβ_40_ and Aβ_42_ peptides in the brain of male and female APP/PS1 mice were not affected by the induction of multifocal cerebral microinfarcts. AD is often accompanied by CAA, which is caused by the deposition of Aβ within the brain arteries. Although Aβ vascular deposition in AD could lead to the blockage of penetrating arterioles resulting in microinfarcts (83–85), the causal effect of multifocal microinfarcts on CAA pathology remains unknown. Here, we show that the frequency of CAA, translated by the number of brain arteries associated with insoluble Aβ aggregates, specifically decreased is the brain of female APP/PS1 mice following induction of microinfarcts. Previous reports have demonstrated that the overall CAA incidence was more important in males in comparison to females (86). Nonetheless, contribution of the microinfarcts to reported effects was not evaluated. Our findings may appear in contrast with previous reports indicating that cerebral hypoperfusion could accelerate CAA, promoting the formation of microinfarcts (87, 88). However, in contrast to our MO model that was used to induce structural lesions, these reports used mild chronic cerebral hypoperfusion in absence of structural injury. The chronic cerebral hypoperfusion models address the role of vascular dysfunction as a risk factor for AD, but do not allow to investigate the impact of sporadic multifocal microinfarcts *per se* on AD pathology progression. Importantly, despite the attenuated burden of Aβ plaques, we found out that the early induction of multifocal microinfarcts altered the recognition memory in male and female WT and APP/PS1 mice, and reduced the state of vigilance, which is associated with an increased disinhibitory behavior, specifically in male APP/PS1 mice in the subacute phase. Interestingly, we found out that the alterations in recognition memory and disinhibitory behavior associated with microinfarct induction were maintained in male APP/PS1 mice, whereas the female APP/PS1 mice seem to recover. These results indicate that microinfarcts worsened the early cognitive deficits of APP/PS1 mice, which were rapid and prolonged in males, but mild and transient in females. It is well established that recognition memory and disinhibitory behavior are progressively altered in AD (52, 56, 89–91). Beside memory impairments, it has been shown that the state of vigilance is reduced during AD-like pathology progression in APP/PS1 mice. As such, our results indicate that multifocal cerebral microinfarct induction accelerated this process at the early stages of AD in young APP/PS1 mice, independently upon Aβ pathology, an effect that was exacerbated in male APP/PS1 mice. Several previous experimental studies have demonstrated that cerebrovascular diseases exacerbate AD-like pathology in various animal models (92–94). However, most of these studies evaluated the consequences of focal cerebral ischemia in animal models in which AD-like pathology is well advanced and outlined an exacerbation Aβ pathology. Our report is the first to investigate the impact of multifocal microinfarcts on AD-like pathology in young APP/PS1 mice, highlighting a sex-dependent acceleration of the disease independently upon Aβ pathology. Our results are in line with previous observations showing that microinfarcts could aggravate early AD pathology in younger patients (45, 46). Nonetheless, our data indicate that this aggravation could occur without necessarily correlating with Aβ pathology, an important aspect that could have major consequences on diagnosis and treatment.

Dysfunction of the cerebrovascular network has been reported in AD patients, and animal models, which is characterized by CBF deregulation as well as BBB disruption (35, 95–98). Several reports indicated that the CBF is reduced in AD leading to chronic hypoperfusion, which deregulates the glucose metabolism required for neuronal functioning (33, 34). Cerebral hypoperfusion associated with CBF reduction has been shown to occur at the early stages of AD even before the appearance of clinical symptoms associated with cognitive decline (99). Moreover, the experimental findings have demonstrated that chronic cerebral hypoperfusion could result in cognitive impairment independently upon Aβ pathology *via* modulation of neuroinflammation (35, 55, 100–107). Nonetheless, contribution of the preclinical neurodegeneration to the reported hypoperfusion cannot be excluded. It has been previously shown that the cerebral hypoperfusion induces cortical microinfarcts, which exacerbate the neurodegenerative cascade and worsen dementia (108). Moreover, CBF reduction could be associated with the accumulation of Aβ in the brain vasculature, which could impair neurovascular coupling (88, 109). Here we show that the induction multifocal microinfarcts caused an acute cerebral hypoperfusion associated with a reduced CBF in the ipsilateral hemisphere in male and female APP/PS1 mice. Importantly, the CBF in the ipsilateral hemisphere was restored in females, whereas in males a chronic hyperperfusion was observed regardless of the presence of Aβ pathology. Following traumatic brain injury (TBI), neurovascular uncoupling associated with an impaired autoregulation of the CBF have been shown to cause in cerebral hyperperfusion, which consequently increased intracranial pressure, subsequently promoting the formation of cytotoxic and vasogenic oedema (110). Interestingly, cerebral hyperperfusion has been previously shown to occur in AD and amnestic mild cognitive impairment (MCI) patients (100). Other reports have demonstrated that the brain regions that are affected early in AD, including the hippocampus, exhibited an increased CBF, which was proposed to be associated with compensatory or pathological increase of neural activity, inflammatory responses, or the secretion vasoactive mediators (111). Our findings indicate that multifocal microinfarcts could have triggered TBI-like mechanisms leading to neurovascular uncoupling, which was more pronounced in males compared to females. Finally, we observed that the microinfarcts caused a chronic dysfunction of the BBB in the brain of APP/PS1 mice independently upon biological sex. BBB dysfunction in AD has been shown to impair glucose transport, increase entry of neurotoxic blood-derived molecules into the brain, exacerbate the inflammatory response, which jointly contribute to neurodegeneration (112). Our results suggest that microinfarcts could contribute to the neurodegenerative cascade by increasing BBB permeability.

Various molecular and cellular mechanisms have been shown to be implicated in regulating the production and clearance of Aβ in the brain (113–115). These include clearance of Aβ *via* specialized vascular receptors such as ABCB1 and LRP1 (113), as well removal by inflammatory cells such as microglia and monocytes (13–15). The clearance of Aβ mediated by ABCB1 and LRP1 across the BBB has been shown to be impaired in AD (113, 116). Here we show that microinfarct induction did not affect the expression of ABCB1 and LRP1 in the brain of male and female APP/PS1. Similarly, the microinfarcts did not influence the expression of BACE1, which is the key enzyme implicated in the pathological cleavage of APP generates Aβ peptides that aggregate overtime into insoluble deposits (81, 117). These results exclude the implication of these molecular mechanisms associated with Aβ production and clearance through the vasculature in the reduced deposition of Aβ in the brain of APP/PS1 mice after microinfarct induction. Microglia have been shown to be critically involved in AD pathology, as impaired microglial cell activity has been linked to a poor Aβ clearance (13). Microglial cell activation in the early phases of AD efficiently clears Aβ and promotes neuronal protection (14, 15), whereas it stimulates the release neurotoxic and pro-inflammatory factors at the advanced stages leading to chronic neuroinflammation and neuronal loss (16, 17). Here we show that multifocal microinfarct induction triggered microglial cell activation and stimulated their phagocytic capabilities at the lesion sites in the brain APP/PS1 mice, independently upon the biological sex, which may account for the attenuated Aβ pathology. This is in line with previous reports showing that stimulation of microglia using immunomodulator molecules increases Aβ clearance *via* phagocytosis (13, 70, 118, 119). However, in our experimental setting, this reduction was not accompanied by enhanced cognitive functions, probably due to the specific pathological effects of microinfarcts independently upon Aβ. Besides microglia, monocytes have been shown to be critically involved in AD (18, 19, 71, 120). Ly6C^high^ CX3CR1^low^ monocytes have been shown to infiltrate the brain and differentiate into microglia-like cells that are actively involved in Aβ removal (14, 71). The generation of Ly6C^low^ CX3CR1^high^ and Ly6C^high^ CX3CR1^low^ monocytes have been reported to be progressively impaired in APP/PS1 mice, correlating with cognitive decline (71). Here we show that microinfarcts increased the frequency of total monocytes in the blood circulation, a response that was attenuated in the presence of Aβ pathology in APP/PS1 mice. Nonetheless, the reduced frequency of monocytes in response to microinfarcts seems to be less potent in female APP/PS1 mice, in which cognitive decline was moderate. Previous reports have demonstrated that monocytes isolated from women possess higher phagocytic capacity and exhibit lower cytotoxic activity in young subjects compared to men (121, 122). Moreover, recent transcriptomic analysis has outlined the presence of sexual dimorphism in immune functions of monocytes, which may be associated with more potent priming of the innate immune functions in females compared to males. Importantly, microinfarct induction promoted the formation of an intermediate Ly6C^int^ monocyte population. It has been previously shown that Ly6C^low^ CX3CR1^high^ monocytes could arise from circulating Ly6C^high^ CX3CR1^low^ monocytes (123). This suggests that the increased frequency of intermediate monocytes in the subacute phase after microinfarct induction could be the result of the transition of Ly6C^high^ CX3CR1^low^ monocytes towards Ly6C^low^ CX3CR1^high^ monocytes (124). CX3CR1^high^ monocytes have been shown to infiltrate the injured tissue to differentiate into regenerative macrophages that promote neuronal protection and repair following excitotoxicity-mediated injury (25). Furthermore, Ly6C^low^ CX3CR1^high^ monocytes play a major role in the vascular Aβ micro-aggregates, attenuating Aβ pathology (18). Moreover, Ly6C^low^ CX3CR1^high^ monocytes have been demonstrated to remove Aβ micro-aggregates from the vasculature (18). To better understand the role of Ly6C^low^ CX3CR1^high^ monocytes, we generated chimeric animals track their recruitment into the brain at different time point dependently upon the biological sex. Ly6C^low^ CX3CR1^high^ monocytes were highly recruited to brain parenchyma in the acute and subacute phases of microinfarct induction but were absent 1 month later, partially expressed microglial marker, and some were found around Aβ plaques. This response seems to be more efficient in female APP/PS1 mice, which may explain the attenuated Aβ pathology as well as cognition recovery specifically in the hippocampus that is involved in episodic memory (19, 122, 125–127). Finally, we found that the frequency of neutrophils increased in both male and female APP/PS1 mice in response to microinfarctions. Neutrophils have been shown to infiltrate the injured brain to support microglia in their repairing processes ​​ (128, 129), which may again explain Aβ pathology attenuation.

Our results indicate that effects mediated by microinfarcts on neurobehavioral outcomes in APP/PS1 mice are independent upon Aβ pathology. As such, we investigated the implication of canonical Wnt pathway, deregulation, which has been reported to play important roles in AD etiology and progression (52, 130). Indeed, expression of DKK1, an endogenous inhibitor of the canonical Wnt pathway, has been shown to be induced in the AD brain, more particularly in neurons near Aβ deposits (131–133). Interestingly, DKK1 has been demonstrated to contribute to synaptic deregulation and neuronal dysfunction, which are exacerbated in presence of Aβ (74, 134). Here we show that microinfarct induction triggered a potent prolonged expression of DKK1 specifically in the brain of male APP/PS1 mice, whereas in females the levels remained low. Importantly, DKK1 expression has been demonstrated to be repressed by estrogens, which provide neuroprotection against ischemic insults in young females, an aspect that is impaired in aged menopaused individuals (135). These results suggest that the excessive induction of DKK1 in the brain of young male APP/PS1 might account for the observed rapid and prolonged cognitive decline despite the reduction of Aβ pathology. DKK1 seems to constitute an interesting factor that is involved in the sexual dimorphism observed following the induction of microinfarcts at the early stages of AD. Future research using aged or young ovariectomized animals is warranted to fully elucidate the role of DKK1 in modulating AD pathology when microinfarcts occur.

## Conclusion

**Figure 11:**
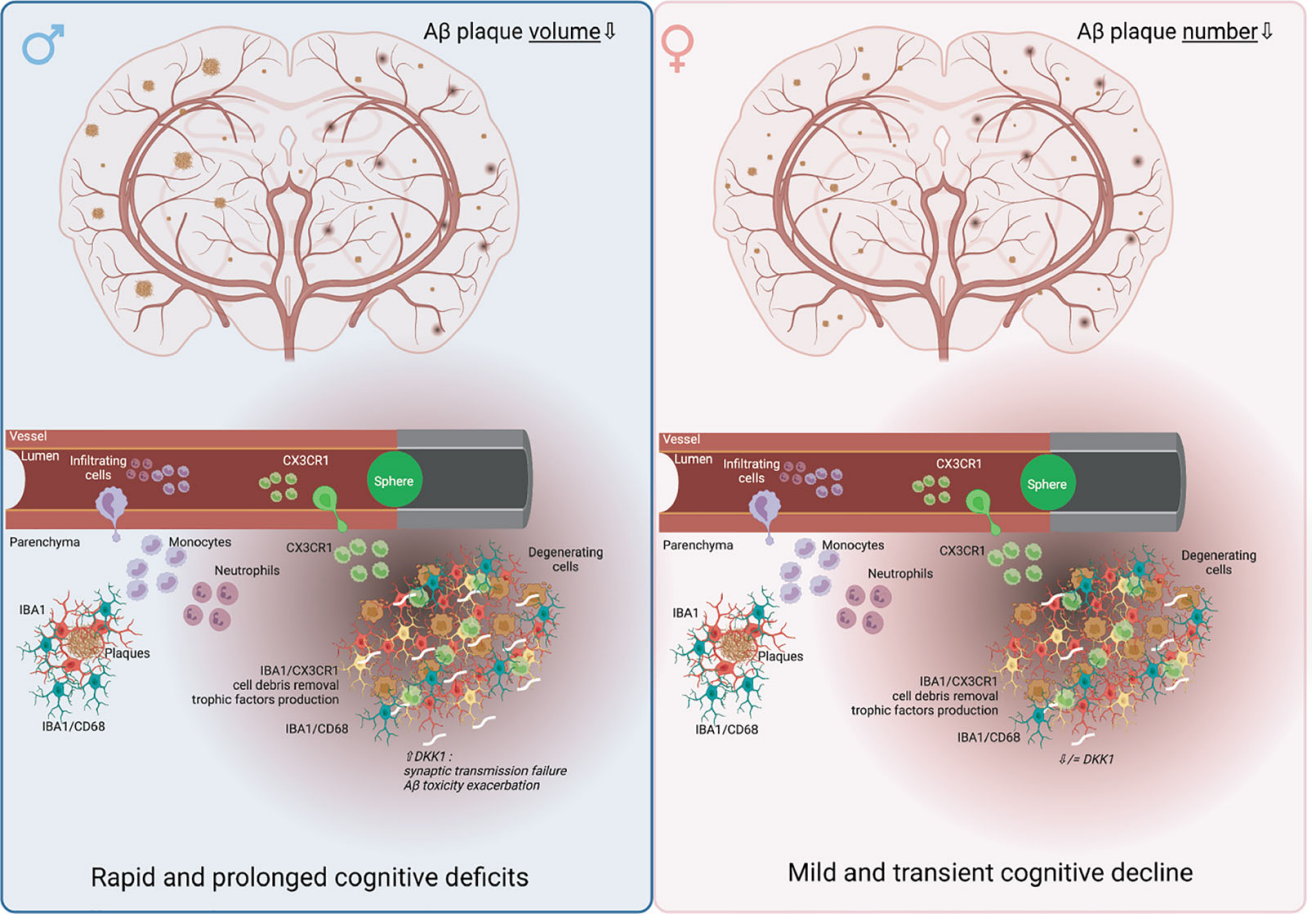
A scheme illustrating impact of multifocal microinfarcts on AD-like pathology in mice. In male APP/PS1 mice, microinfarcts decrease the volume of Aβ plaques, which could be associated with the activation of microglial cells and the moderate recruitment of peripheral CX3CR1^high^ monocytes at the lesion site and Aβ plaques. However, DKK1 expression increases at the lesion sites, presumably contributing to the rapid and prolonged cognitive deficits. In females, microinfarcts decrease the number of Aβ plaques, which could be associated with the potent activation of microglia and the important recruitment of peripheral CX3CR1^high^ monocytes at the lesion site and Aβ plaques. DKK1 expression, which is repressed by estrogens, remains low at the lesion sites, presumably leading to mild and transient cognitive decline. Created with BioRender.com.

Our study provides new insights into sex-dependent effects of multifocal cerebral microinfarcts on AD-like pathology in young APP/PS1 mice (**Figure 11**). Interestingly, we reported here a previously undescribed impact of biological sex on Aβ dynamics. Young male APP/PS1 mice developed less plaques than age-matched females, but of higher volume, similarly impacting cognitive functions at the early stages of AD-like pathology. Surprisingly, multifocal microinfarcts reduced Aβ deposition in the brain in male and female APP/PS1, which was more pronounced in females, while exacerbating cognitive decline more importantly in males. The effects on Aβ could be explained by an enhanced microglial activity and recruitment of peripheral phagocytic cells into the brain, thus potentially promoting Aβ clearance. Our findings advocate for independent but synergistic effects of multifocal microinfarcts and Aβ neuropathology in exacerbating cognitive decline, which was rapid and prolonged in young APP/PS1 males, but mild and transient in females (**Figure 11**). DKK1 is excessively induced in the brain of male APP/PS1 mice in response to focal microinfarcts, while its expression remains low in the brain of age-matching females. DKK1 contributes to AD pathology by causing synaptic failure and by mediating as well as exacerbating Aβ-mediated neuronal dysfunction and loss. DKK1 might constitute the pathological link that independently but synergistically mediates the effects of multifocal microinfarcts and Aβ neuropathology on cognitive decline. AD affects more women than men in the elderly population, while the younger female population seems to be protected, mainly due to the presence of high levels of estrogens. However, this protection in females seems to be completely reversed in the post-menopausal stage. DKK1 expression is negatively regulated by estrogens, highlighting its role in the sexual dimorphism observed in AD-like pathology modulation in APP/PS1 mice in response to multifocal microinfarcts. Taken together, DKK1 seem to constitute a promising target for the development of novel biological sex-tailored therapeutic interventions. Finally, our study indicates that the major sex-dependent effects of multifocal microinfarcts, which were reported to be present in 42% of AD, should be taken into consideration while proving diagnosis and prognosis or developing therapeutic strategies, especially those targeting Aβ.

## Data Availability Statement

The original contributions presented in the study are included in the article/supplementary material. Further inquiries can be directed to the corresponding author.

## Ethics Statement

The animal study was reviewed and approved by Laval University Animal Welfare Committee.

## Author Contributions

All authors have approved the final version of the manuscript and confirm that all relevant data are included in the paper. The authors declare no conflict of interest and contributed as follows. SL: Conceptualization, experimentation, data interpretation, drafting, and editing. VP: Flow cytometry experiment and analysis, blood sample preparation and editing. SR: Editing. AEA: Conceptualization, experimentation, data interpretation, drafting and editing.

## Funding

This work is supported by grants from the Scottish Rite Charitable Foundation of Canada (SRCFC) (17106), the Natural Sciences and Engineering Research Council of Canada (NSERC) (RGPIN-2017-06119), the Heart and Stroke Foundation of Canada (HSFC) (G-18-0022118), and the Canadian Institutes of Health Research (CIHR) (169062) (all to AEA). AEA holds a Tier 2 Canada Research Chair in molecular and cellular neurovascular interactions. SR holds a CIHR Tier 1 Canada Research Chair in neuroimmunology and a CIHR foundation scheme grant.

## Conflict of Interest

The authors declare that the research was conducted in the absence of any commercial or financial relationships that could be construed as a potential conflict of interest.

## Publisher’s Note

All claims expressed in this article are solely those of the authors and do not necessarily represent those of their affiliated organizations, or those of the publisher, the editors and the reviewers. Any product that may be evaluated in this article, or claim that may be made by its manufacturer, is not guaranteed or endorsed by the publisher.

## Glossary

Aβ: Amyloid-β
ABCB1: ATP Binding Cassette Subfamily B Member 1
ACK: Ammonium
Chloride: Potassium
AD: Alzheimer’s Disease
APP/PS1: Amyloid Precursor Protein/Presenilin 1
BACE1: beta-site amyloid precursor protein cleaving enzyme-1
BBB: Blood Brain Barrier
BSA: Bovine Serum Albumin
CAA: Cerebral Amyloid Angiopathy
CBF: Cerebral Blood Flow
CCA: Common Carotid Artery
CD: Cluster of Differentiation
CSVD: Cerebral Small Vessel Disease
CX3CR1: C-X3-X motif Chemokine Receptor 1
DAB: Diaminobenzidine Tetrahydrochloride
DAPI: 4′,6-diamidino-2-phenylindole
DKK1: Dicckopf-1
DPBS: Dulbecco’s phosphate-buffered saline
DPX: Dibutylphtalate Polystyrene Xylene
ECA: External Carotid Artery
ECL: Enhanced Chemiluminescence
EGFP: Enhanced Green Fluorescent Protein
EthO: Ethyl alcohol
FBS: Fetal Bovine Serum
FISH: RNAscope™ Fluorescent *in situ* hybridization
IBA1: Ionized Calcium Binding Adaptor molecule 1
IgG: Immunnoglobulin G
KPBS: Potassium Phosphate-Buffered Saline
LSCI: Laser Speckle Contrast Imaging
LRP1: LDL Receptor Related Protein 1
MCI: Mild Cognitive Impairments
MO: micro-occlusion
NFTS: Neurofibrillary Tangles
NGS: Normal Goat Serum
NIR: Near-Infra Red
NOR: Novel Object Recognition
OF: Open Field
PPA: Pterygopalatine Artery
PFA: Paraformaldehyde
PB: Phosphate Buffer
PVDF: Polyvinylidene Fluoride
ROI: Region of Interest
SDS: Sodium Dodecyl Sulphate
SEM: Standard Error of the Mean
TBI: Traumatic Brain Injury
TBS: Tris-Buffered Saline
TBS-T: Tris-Buffered Saline – Tween 20
WT: Wildtype
